# Genetic relevance and determinants of mitral leaflet size in hypertrophic cardiomyopathy

**DOI:** 10.1186/s12947-019-0171-1

**Published:** 2019-10-28

**Authors:** Hyemoon Chung, Yoonjung Kim, Chul-Hwan Park, Jong-Youn Kim, Pil-Ki Min, Young Won Yoon, Tae Hoon Kim, Byoung Kwon Lee, Bum-Kee Hong, Se-Joong Rim, Hyuck Moon Kwon, Kyung-A Lee, Eui-Young Choi

**Affiliations:** 1 Division of Cardiology, Department of Internal Medicine, Kyung Hee University School of Medicine, 23 Kyung Hee Dae-ro, Seoul, Dongdaemun-gu 02447 Republic of Korea; 2 Department of Laboratory Medicine, Gangnam Severance Hospital, Yonsei University College of Medicine, Seoul, South Korea; 3 Department of Radiology, Gangnam Severance Hospital, Yonsei University College of Medicine, Seoul, South Korea; 4 Division of Cardiology, Heart Center, Gangnam Severance Hospital, Yonsei University College of Medicine, 211 Eonju-Ro, Gangnam-Gu, Seoul, 06273 South Korea

**Keywords:** Hypertrophic cardiomyopathy, Mitral valve, Mitral leaflet, Gene

## Abstract

**Background:**

Whether mitral leaflet elongation is a primary phenotype of hypertrophic cardiomyopathy (HCM) is controversial. We investigated the genetic relevance and determinants of mitral leaflet size by performing extensive gene analyses in patients with HCM.

**Methods:**

Anterior mitral leaflet (AML) lengths were measured in HCM patients (*n* = 211) and age- and sex-matched controls (*n* = 30) using echocardiography with hemodynamic and chamber geometric assessments. We analyzed 82 nuclear DNA (8 sarcomeric genes, 74 other HCM-associated genes) and mitochondrial DNA. Cardiac magnetic resonance imaging (CMR) was performed in the 132 HCM patients.

**Results:**

Average indexed AML was significantly longer for HCM than for controls (17.2 ± 2.3 vs. 13.3 ± 1.6 mm/m^2^, *P <  0.001*). Average AML length correlated with body surface area (BSA), left ventricular (LV) end-systolic volume (*P <  0.001*) and LV mass by CMR *(P < 0.001)*. Average indexed AML by BSA of pure-apical HCM was significantly shorter than other typed HCM (16.6 ± 2.0 vs. 17.4 ± 2.4 mm/m^2^, *P = 0.025*). Indexed AML was independently correlated with left atrial wall stress. The thin filament mutation group showed larger average AML (31.9 ± 3.8 vs. 29.6 ± 3.8 mm, *P = 0.045*), but this was not significant with the indexed value. No difference in AML size among subgroups was observed based on the presence of sarcomere protein or mitochondria-related gene variants (*P > 0.05*).

**Conclusion:**

AML elongation was a unique finding of HCM. However, the leaflet size was more related to chamber geometry and hypertrophy pattern rather than genetic factors within overt HCM.

## Introduction

Mitral leaflet elongation is related to obstructive hypertrophic cardiomyopathy (HCM) [1]. Although, there are still some controversies whether classical pathogenic sarcomere gene variants independently affect mitral leaflet enlargement [2–4]. Previous basic studies have shown that some sarcomere genes affect valvular growth [5, 6]. In addition, a recent study further confirmed that leaflet elongation is a primary phenotype in subclinical HCM patients with pathogenic sarcomere gene mutations [7]. However, these investigations mainly focused on variants in classical pathogenic sarcomere genes such as *MYBPC3* and *MYH7*, regardless of the morphologic phenotype of the hypertrophy pattern [2–4, 7]. The contributions of various gene variant groups other than sarcomere genes have not been extensively investigated. In addition, about 40–60% of patients with overt HCM do not have any pathogenic sarcomere gene mutations [4]. Furthermore, regarding mitral leaflet size, there was no comparison study between pathogenic variant group and non-variant group within overt hypertrophy patients. Therefore, it is still unclear whether mitral leaflet size in overt HCM is mainly determined by genotype or by additional geometric or hemodynamic factors. In this study, to reveal the genetic relevance and effects of LV geometric change to mitral leaflet size, an extensive HCM gene panel comprising 82 nuclear DNA (nDNA) genes (8 sarcomere genes, 74 other HCM-associated genes) and mitochondrial DNA (mtDNA) genes was analyzed along with diverse morphologic phenotype definitions and hemodynamic factors.

## Methods

### Study population

A total of 432 patients treated at a single center were enrolled in an HCM Registry from 2006 to 2014. Among them, 220 patients were excluded due to insufficient data, follow-up loss, or declining study enrollment. Of these patients, one had poor image quality that precluded measuring mitral leaflet size in any view. Finally, 211 patients underwent genetic testing. The patients enrolled in the study had maximal left ventricular (LV) hypertrophy greater than 13 mm and a ratio of maximal thickness to posterior wall thickness greater than 1.3 without an underlying cause of hypertrophy, such as uncontrolled hypertension or aortic stenosis. Cases of abnormal papillary muscle insertion to the apex with thickening were also included. Patterns of LV hypertrophy were classified as apical HCM (ApHCM) and non-ApHCM (asymmetrical hypertrophy, diffuse hypertrophy, and focal segmental hypertrophy). ApHCM was classified as pure apical type (hypertrophy confined below the papillary muscle level) and mixed type (mixed pure apical and asymmetrical septal hypertrophy but maximal thickness in the apex) according to echocardiographic findings. All patients underwent screening analysis for Fabry disease and were confirmed negative for the galactosidase alpha (GLA) variant. For comparison, anterior mitral leaflet (AML) size was measured in 30 age- and sex-matched controls. The study protocol was approved by our institutional review board (3–2015-0019), and written informed consent was obtained for each subject.

### Genetic testing and analysis

#### HCM gene panel (nDNA) design

**Fig. 1:**
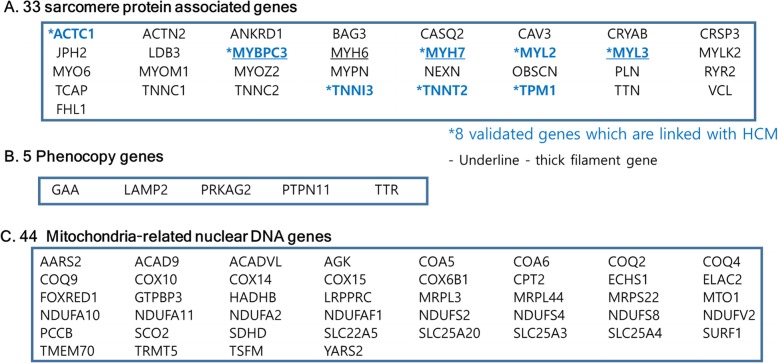
The comprehensive hypertrophic cardiomyopathy (HCM)-specific panel consisted of 82 nuclear genes including (**a**) 33 sarcomere-associated genes, (**b**) 5 phenocopy genes, and (**c**) 44 mitochondria-related nuclear DNA (nDNA) genes

A literature search of the PubMed database was performed for targeted gene selection for the comprehensive HCM-specific panel. It included 82 nDNA genes (33 sarcomere protein genes, 5 phenocopy genes, and 44 nuclear genes linked to mitochondrial cardiomyopathy, Fig. 1 and Additional file 2 Table S1). HCM genes consisted of 8 validated sarcomere genes and 25 putative HCM genes [8].

#### DNA preparation

The details are described at Additional file 1 Method S1.

#### Library construction and sequencing of the HCM gene panel and mtDNA

The details are described at Additional file 1 Method S2.

#### Identification of potential pathogenic mtDNA variants

Non-haplogroup-associated novel and rare variants were evaluated for their potential pathogenicity based on variant location, amino acid change, and evolutionary conservation [9].

#### Data analysis of the HCM gene panel

The Burrows-Wheeler aligner algorithm with default parameters was used to align reads to the human reference genome sequence GRCh37 [10]. SAMTools was used to convert the sequence alignment map file to the BAM format [11]. Sorting and removal of duplications were performed using the Picard tool (http://broadinstitute.github.io/picard/). GATK was used to perform indel realigning and base-quality score re-calibration [12]. Variants were annotated with ANNOVAR [13] and Variant Effect Predictor (http://asia.ensembl.org/info/docs/tools/vep/index.html). Only bases meeting the minimum base quality (≥ 20) from reads are considered. Variants were further filtered with altered allele frequency > 30%, 50× coverage, and population frequency < 0.01 in the 1000 Genome Project, ESP6500, ExAC databases, and the Korean Reference Genome Database (http://152.99.75.168/KRGDB), which was constructed with whole-genome sequencing data from 1100 Korean individuals. Prediction of the potential pathogenicity was performed using Alamut® Visual software (Interactive Biosoftware, Rouen, France), Human Gene Mutation Database professional version, release 2016.1 (http://www.hgmd.org/), ClinVar, and the Atlas of Cardiac Genetic Variation. The impact of missense change was predicted with Align GVGD, SIFT, PolyPhen and MutationTaster. Visual inspection of candidate variants were performed using the Integrative Genomics Viewer (IGV) [14] and we performed Sanger confirmation of pathogenic variants which showed unacceptable quality metrics. Variants were classified based on the American College of Medical Genetics and Genomics standards and guidelines [15, 16].

#### Data analysis of the mitochondrial genome

The details are described in Additional file 1 Method S3.

### Echocardiographic analysis

Comprehensive echo-Doppler evaluation was performed according to current American Society of Echocardiography guidelines [17]. A routine standard echocardiography study was performed to measure systolic and diastolic parameters as follows: LV end-diastolic volume and LV end-systolic volume (LVESV) were measured with the biplane Simpson’s method, and LV ejection fraction was calculated. Left atrial (LA) volume was measured at the end-systole by the ellipsoidal method, and LA volume index was calculated as LA volume/body surface area (BSA). Peak early (E) and late (A) diastolic mitral inflow velocities were measured in apical four-chamber view. Tissue Doppler interrogation was performed in the septal mitral annulus in apical four-chamber view, followed by measurement of the peak systolic mitral annulus velocity (s′) and early diastolic mitral annulus peak velocity (e′). The ratio of E/e′ was calculated. LV wall thickness was measured in all cross-sectional planes. Continuous wave Doppler was used to measure peak velocity across the LV outflow tract (LVOT), and the pressure gradient was calculated using the Bernoulli equation as follows: 4 × (peak velocity across the LVOT) [18]. It was measured at resting and during Valsalva maneuver. LVOT obstruction was defined as a systolic pressure gradient ≥30 mmHg. Contrast echocardiography was performed in patients with a poorly defined LV border. Mitral regurgitation (MR) degree was defined as trivial, I, II, III, or IV according to regurgitation area.

### Cardiac magnetic resonance imaging (CMR)

CMR was performed using a 1.5-T scanner (Magnetom Avanto®; Siemens Medical Solutions, Erlangen, Germany) with a phased array body coil. The LV 2-, 3-, 4-chamber, and short axis views were obtained using cine images with steady-state free precession sequence. The endocardial and epicardial borders were contoured using a semi-automated method (Argus®; Siemens, Germany or Qmass® MR 8.1; Medis, Leiden, the Netherlands); subsequently, the LVEDV and LVESV were measured. To determine the end-diastolic LV mass, the difference between the epicardial and endocardial areas for all slices was multiplied by the slice thickness and section gap, and then multiplied by the specific gravity of the myocardium (1.05 g/mL). Papillary muscle mass was included in the LV cavity and excluded from the LV mass measurements.

### Mitral leaflet length measurement

**Fig. 2:**
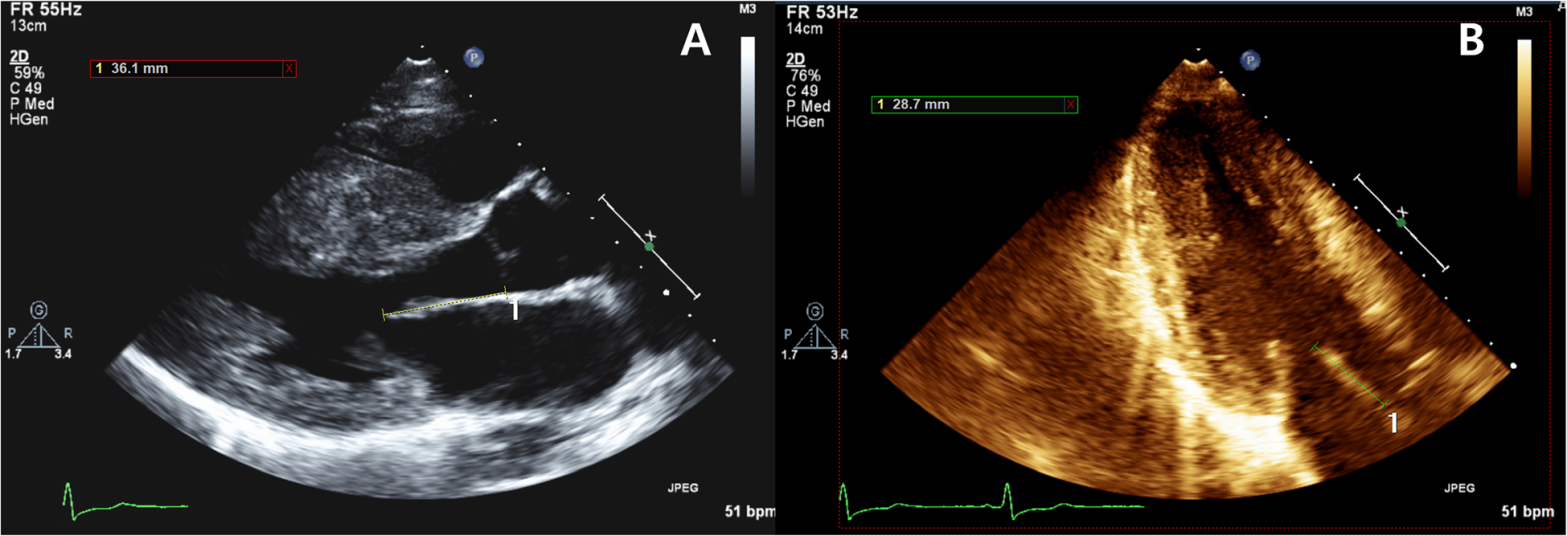
Schematic illustration of anterior mitral leaflet distance measurement in the parasternal long axis view (**a**) and apical 3-chamber view (**b**)

AML lengths were measured in the parasternal long axis (PLX) and apical three-chamber (3CH) views, the same measurements from age- and sex-matched controls were taken for comparison. In both views, AML lengths were measured in diastole, with the leaflets maximally extended and parallel to the anterior septum, and defined as the distance from the most distal extent of the anterior leaflet to its insertion into the posterior aortic wall (Fig. 2). The measurements were repeated in 10 randomly selected patients to assess reproducibility.

### LV geometry and mitral valve wall stress assessment

For end-systolic mitral valve wall stress assessment, the index from LV side was roughly calculated as (LV end-systolic pressure) × LVESV, where LV end-systolic pressure was calculated as aorta end-systolic pressure (2 × systolic blood pressure + diastolic blood pressure/3) + peak systolic LVOT pressure gradient. E/e′ × LA volume was used for measurement of diastolic mitral valve wall stress index from the LA side (LAWS).

### Statistical analysis

Continuous variables with normal distributions are reported as the mean ± standard deviation or 95% confidence interval. Student’s *t*-tests were used to compare the means of continuous variables that were approximately normally distributed between the two groups. Normality was determined using the Shapiro-Wilk test. Categorical variables are reported as counts (or percentages) and were compared using chi-square tests. For comparison of more than two groups, analysis of variance was performed with post-hoc analysis (LSD) for subgroup comparison. For the reproducibility test, paired sample t-tests and Pearson’s correlation coefficients were determined. All clinical statistical analyses were performed using SPSS version 23.0 statistical package (IBM Corp., Armonk, NY, USA). A two-sided *p*-value < 0.05 was considered statistically significant.

## Results

### Baseline characteristics

**Fig. 3:**
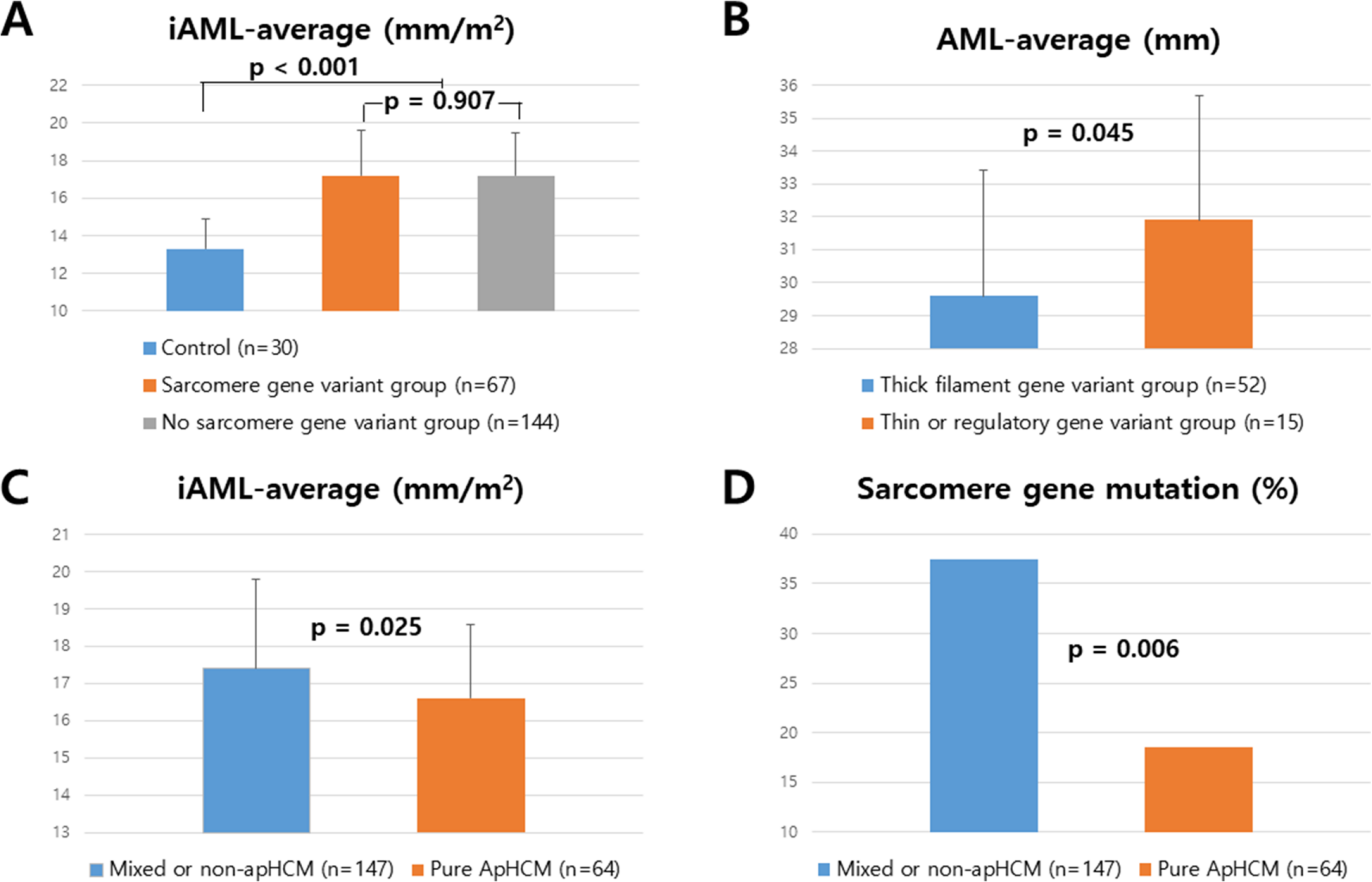
Comparison of indexed AML between sarcomere gene mutation positive and mutation negative patients along with comparison to control (**a**). Comparison of AML-average between thick sarcomere gene variants and thin or regulatory sarcomere gene variants (**b**). Comparison of indexed AML (**c**) and prevalence of sarcomere gene variants (**d**) between pure apical HCM and mixed or non-apical HCM. AML, anterior mitral leaflet length; iAML, indexed AML, HCM, hypertrophic cardiomyopathy; bar represents mean ± standard error

The mean age of patients was 59 ± 14 years, and 63 (30%) were female. Of them, 49 (23%) had obstructive HCM; 100 patients (47%) had ApHCM, and 64 (64%, 64/100) of these patients had pure-type ApHCM. The AML lengths of patients with HCM, measured in both PLX and 3CH, were significantly longer than those of controls (32.1 ± 4.5 vs. 26.4 ± 2.9 mm in PLX; 28.3 ± 3.6 vs. 23.6 ± 3.1 mm in 3CH; both *P < 0.001*). Even after being indexed by BSA, patients with HCM had longer AMLs than control (18.3 ± 2.8 vs. 14.0 ± 2.0 mm/m^2^ in PLX; 16.1 ± 2.4 vs. 12.5 ± 1.8 mm/m^2^ in 3CH, *P < 0.001*) (Fig. 3).

### Genetic characteristics

Based on the American College of Medical Genetics and Genomics guideline, [16] 67 of 211 (31.8%) cases had 71 pathogenic or likely pathogenic variants in 33 sarcomere-associated genes (33 *MYBPC3*, 19 *MYH7*, 14 *TNNI3*, 2 *MYH6*, 1 *JPH2*, 1 *TNNC1*, and 1 *MYL3*). Four patients harbored more than one variant in HCM genes. We identified homozygous or compound heterozygous variants in *MYBPC3* in one patient and co-variants in three patients (two had *MYBPC3* and *MYH7*, and one had *MYBPC3* and *JPH2*). Twenty-seven (13%) patients had probably damaging mtDNA variants, 15 (7%) had mitochondria-related nDNA variants, and 1 had a pathogenic variant in GAA (Additional file 2 Table S2 and Table S3). Patients with ApHCM had a lower prevalence of sarcomere protein gene variants (41.4% vs. 20.8%, *P = 0.001*) than patients with non-ApHCM.

### Correlation with mitral leaflet size

We observed significant correlations between average AML length and BSA (r = 0.329, *P < 0.001*), LV end-diastolic volume, LVESV (r = 0.341, *P < 0.001*), LV mass by CMR (r = 0.338, *P < 0.001*), LV end-systolic wall stress index, LA volume, and maximal thickness. Of these parameters, BSA showed significant and independent correlation with average AML length (β = 0.292, *P < 0.001*); hence, AML lengths indexed by BSA (iAML) were used for further analyses. The average iAML of pure ApHCM (*n* = 64) was significantly shorter than that of mixed or non-ApHCM (*n* = 147; 16.6 ± 2.0 vs. 17.4 ± 2.4 mm/m^2^, *P = 0.025*) along with a lower prevalence of sarcomere protein gene mutation (19% vs. 36%, *P = 0.006*) (Table 1 and Fig. 3). Although the iAML of obstructive HCM was not significantly higher than non-obstructive HCM (17.8 ± 2.9 vs. 17.0 ± 2.1 mm/m^2^, *P = 0.103*), iAML was significantly correlated with the LVOT peak pressure gradient (resting and during Valsalva maneuver, r = 0.254, *P = 0.001*). Average iAML was significantly correlated with age, LA volume, LAWS (r = 0.275, *P < 0.001*), MR grade (r = 0.373, *P < 0.001*), and E/e′. In multivariable analysis, LAWS was independently associated with the average iAML (β = 0.212, *P = 0.012*) (Table 1). The relationships between AML size and echocardiographic parameters remained significant even after exclusion of pure-type ApHCM.

**Table 1:** Comparison between sarcomere protein gene variant group and non-variant group

	Total (*n* = 211)			Sarcomere protein gene variant group (*n* = 67)		
	Presence of sarcomere protein gene variant group (*n* = 67)	Absence of sarcomere protein gene variant group (*n* = 144)	P	Thick filament gene variant group (*n* = 52)	Thin filament or regulatory gene variant group (*n* = 15)	P
Age, years	54.8 ± 14.3	61.3 ± 12.8	0.001	55.3 ± 13.0^**^	52.9 ± 18.5^*^	0.557
Women, n (%)	25 (37)	38 (26)	0.106	23 (44)^*^	2 (13)^††^	0.029
Hypertension, n (%)	28 (42)	91 (63)	0.004	22 (42)^**^	6 (40)^**^	0.873
Diabetes, n (%)	12 (18)	27 (19)	0.884	9 (17)	3 (20)	0.811
Body surface area, m^2^	1.76 ± 0.18	1.78 ± 0.21	0.596	1.74 ± 0.19	1.82 ± 0.13	0.135
FHx of SCD-1st, n (%)	6 (9)	8 (6)	0.356	4 (8)	2 (13)	0.500
Syncope, n (%)	6 (9)	4 (3)	0.049	6 (12)^*^	0 (0)^†^	0.168
5-year SCD risk, % (*n* = 123)	2.64 ± 1.51	2.01 ± 1.66	0.040	2.56 ± 1.38	2.88 ± 1.92	0.538
Echocardiography and CMR						
ApHCM, n (%)	21 (31)	79 (55)	0.001	13 (25)^**^	8 (53)^††^	0.037
LVOT PPG (rest), mmHg	9.9 ± 11.6	15.0 ± 23.3	0.041	10.1 ± 12.4	9.5 ± 8.7	0.874
LVOT PPG (Valsalva), mmHg	15.9 ± 19.2	28.2 ± 37.5	0.005	16.7 ± 20.8	12.8 ± 11.0	0.556
Dynamic obstruction, n(%)	13 (19)	36 (25)	0.370	12 (23)	1 (7)	0.157
LVEDV, mL	70.6 ± 28.1	66.5 ± 21.9	0.252	71.1 ± 28.7	68.7 ± 26.6	0.770
LVESV, mL	25.4 ± 12.0	23.1 ± 8.8	0.106	26.1 ± 12.9	23.0 ± 7.9	0.374
LA volume index, mL/m^2^	41.0 ± 22.0	34.8 ± 15.0	0.042	39.5 ± 19.1	46.1 ± 30.4^*^	0.307
MR grade	0.52 ± 0.35	0.47 ± 0.26	0.216	0.51 ± 0.39	0.57 ± 0.18	0.585
LV ejection fraction, %	63.8 ± 7.3	64.9 ± 5.8	0.239	63.0 ± 7.8	66.6 ± 4.5	0.096
s′, cm/s	6.6 ± 1.6	6.8 ± 1.8	0.408	6.6 ± 1.6	6.6 ± 1.9	0.905
E/e′	15.4 ± 6.7	14.6 ± 5.5	0.371	15.2 ± 6.6	16.3 ± 7.2	0.571
Maximal thickness, mm	19.7 ± 3.7	18.6 ± 3.4	0.035	20.1 ± 3.5^**^	18.1 ± 4.1^††^	0.061
LV mass index by CMR, g/m^2^ (*n* = 132)	88.0 ± 21.1	84.9 ± 23.9	0.458	83.8 ± 18.3	102.9 ± 24.3^*^	0.014
AML lengths						
AML-PLX, mm	31.4 ± 4.6	32.5 ± 4.4	0.109	30.8 ± 4.5^*^	33.5 ± 4.6	0.046
AML-3CH, mm	28.7 ± 3.9	28.1 ± 3.4	0.230	28.4 ± 3.9	30.0 ± 3.9	0.187
AML-average, mm	30.1 ± 3.9	30.2 ± 3.5	0.848	29.6 ± 3.8	31.9 ± 3.8	0.045
iAML-PLX, mm/m^2^	18.0 ± 2.8	18.5 ± 2.8	0.222	17.8 ± 2.8	18.4 ± 2.6	0.453
iAML-3CH, mm/m^2^	16.5 ± 2.4	16.0 ± 2.3	0.164	16.5 ± 2.6	16.5 ± 1.7	0.941
iAML-average, mm/m^2^	17.2 ± 2.4	17.2 ± 2.3	0.907	17.1 ± 2.5	17.5 ± 1.9	0.599

Thick filament genes include *MYH7*, *MYBPC3*, *MYH6*, and *MYL3*. *p < 0.05, **p < 0.01 versus absence of sarcomere gene variant group. *AML* anterior mitral leaflet, *PLX* parasternal long axis view, *3CH* three chamber view, *iAML* indexed AML, *FHx* family history, *SCD-1st* sudden cardiac death of 1st degree relatives, *LV* left ventricular, *LVEDV* LV end-diastolic volume, *LVESV* LV end-systolic volume, *LVESWS* LV end-systolic wall stress, *LA* left atrial, *LVOT* LV outflow tract, *PPG* peak pressure gradient, *MR* mitral regurgitation, *s’* peak systolic septal mitral annular velocity, *E/e’* ratio of early mitral inflow and annular velocity, *CMR* cardiac magnetic resonance imaging †p < 0.05, ††p < 0.01 versus thick sarcomere gene mutation group

### Genetic relevance to mitral leaflet length

No significant difference was observed in iAML lengths between the patients with and without pathogenic sarcomere protein gene variants. This non-significance remained after exclusion of patients with pure-type ApHCM. Among patients with sarcomere protein gene variants, there was no significant difference between the *MYBPC3* (*n* = 32) and *MYH7* (*n* = 17) groups (average iAML, 16.8 ± 2.6 vs. 17.8 ± 2.2 mm/m^2^, *P = 0.184*). The *MYBPC3* mutation group has shorter AML-PLX values (32.4 ± 4.4 vs. 30.6 ± 5.0 mm, *P = 0.042*), but iAML was not significantly different. The sarcomere protein gene variant group was further divided into thick filament gene variant (*n* = 52) and thin filament or regulatory gene variant (*n* = 15) groups. The thin filament group showed larger AML size (31.9 ± 3.8 vs. 29.6 ± 3.8 mm/m^2^, *P = 0.045* with average AML), but this was not significant with the indexed value (*P > 0.05*) (Table 2). No significant difference in mitral leaflet size was observed upon classification of patients into non-variant, only mitochondrial-related variant, only sarcomere protein gene variant, and both sarcomere and non-sarcomere gene variant groups (*P > 0.05*) (Table 3).

**Table 2:** Univariate and multivariable analysis of correlation for anterior mitral leaflet lengths

	Univariate analysis			Multivariable	Univariate analysis			Multivariable
	AML-PLX (r)	AML-3CH (r)	AML-average (r)	AML-average (β, *p* value)	iAML-PLX (r)	iAML-3CH (r)	iAML-average (r)	iAML-average (β, p value)
Age	−0.123	− 0.221^**^	− 0.198^**^		0.145^*^	0.081	0.117	
BSA	0.299^**^	0.281^**^	0.329^**^	0.292 (< 0.001)				
LVEDV	0.246^**^	0.297^**^	0.315^**^					
LVESV	0.257^**^	0.340^**^	0.341^**^	0.207 (0.002)				
LVESWS	0.239^**^	0.323^**^	0.317^**^					
LV ejection fraction	−0.118	−0.190^**^	−0.164^*^					
LA volume	0.132	0.197^**^	0.174^*^		0.124	0.189^**^	0.180^**^	
Maximal thickness	0.146^*^	0.225^**^	0.189^**^					
LVOT-PPG (Resting)	0.039	0.044	0.033		0.244^**^	0.225^**^	0.230^**^	
LVOT-PPG (Valsalva)	0.050	0.044	0.047		0.248^**^	0.240^**^	0.254^**^	0.110 (0.203)
MR grade	0.072	0.181^**^	0.147^*^	0.201 (0.004)	0.280^**^	0.390^**^	0.373^**^	0.146 (0.069)
E/e’	0.023	−0.049	−0.007		0.238^**^	0.188^**^	0.239^**^	
LA wall stress	0.079	0.071	0.096		0.242^**^	0.243^**^	0.275^**^	0.212 (0.012)
CMR findings (*n* = 132)								
LV mass	0.301^**^	0.289^**^	0.338^**^	0.227 (0.013)†				
LVEDV	0.156	0.365^**^	0.291^**^					
LVESV	0,104	0.309^**^	0.228^**^					

^*^p < 0.05, ^**^p < 0.01, †adjustment for BSA, MR grade, LVEDV and LVESV by CMR; *BSA* body surface area, *LVESWS* LV end-systolic wall stress. See abbreviations in Table 1

**Table 3:** Clinical, anatomic, and hemodynamic characteristics according to the presence of genetic variant

	No pathogenic or likely pathogenic variant group (*n* = 110)	^1^Only mitochondria related nDNA or mtDNA variant group (*n* = 33)	^1^Only sarcomere gene variant group (*n* = 58)	^1^Both sarcomere and mitochondria-related gene variant group (*n* = 9)	^§^P
Age, years	61.1 ± 12.9	61.5 ± 12.3	54.4 ± 14.7^**, †^	57.0 ± 11.8	0.014
Women, n (%)	31 (28)	6 (18)	21 (36)	4 (44)	0.227
Hypertension, n (%)	69 (63)	21 (64)	26 (45)	2 (22)	0.019
Diabetes, n (%)	21 (19)	6 (18)	10 (17)	2 (22)	0.982
Body surface area, m^2^	1.78 ± 0.21	1.79 ± 0.20	1.77 ± 0.19	1.70 ± 0.11	0.679
FHx of SCD-1st, n (%)	6 (6)	2 (6)	5 (9)	1 (11)	0.820
Syncope, n (%)	3 (3)	1 (3)	5 (9)	1 (11)	0.270
5-year SCD risk, % (n = 123)	2.01 ± 1.72	2.02 ± 1.45	2.73 ± 1.58^*^	2.10 ± 0.97	0.163
Echocardiography and CMR					
ApHCM, n (%)	57 (52)	22 (67)	19 (33)	2 (22)	0.004
LVOT PPG (rest), mmHg	17.4 ± 26.5	8.0 ± 7.4^*^	10.5 ± 11.9	5.2 ± 2.1	0.005
LVOT PPG (Valsalva), mmHg	31.2 ± 40.7	17.4 ± 23.4	18.5 ± 21.1^*^	6.2 ± 2.9	0.025
Dynamic obstruction, n (%)	31 (28)	5 (15)	13 (22)	0 (0)	0.142
LVEDV, mL	66.8 ± 22.5	66.2 ± 20.6	69.9 ± 27.1	71.3 ± 34.1	0.730
LVESV, mL	22.5 ± 8.4	24.8 ± 9.9	24.6 ± 10.2	29.2 ± 20.3	0.145
LA volume index, mL/m^2^	35.2 ± 14.9	31.8 ± 11.1	40.6 ± 22.9†	43.6 ± 15.7	0.051
MR grade	0.49 ± 0.28	0.40 ± 0.21	0.52 ± 0.36	0.56 ± 0.17	0.217
LV ejection fraction, %	65.7 ± 5.0	62.5 ± 7.6^*^	64.3 ± 6.9	61.2 ± 8.7^*^	0.021
s′, cm/s	6.8 ± 1.7	6.9 ± 2.1	6.6 ± 1.7	7.0 ± 1.4	0.748
E/e′	14.8 ± 5.5	13.9 ± 5.3	15.6 ± 6.9	13.5 ± 4.6	0.462
Maximal thickness, mm	18.7 ± 3.6	18.1 ± 2.6	19.6 ± 3.8†	19.8 ± 3.5	0.164
LV mass index by CMR, g/m^2^ (*n* = 132)	85.1 ± 23.9	84.3 ± 24.3	88.5 ± 22.0	84.7 ± 15.9	0.885
AML lengths					
AML-PLX, mm	32.4 ± 4.5	32.5 ± 4.3	31.7 ± 4.7	29.7 ± 3.7	0.260
AML-3CH, mm	28.2 ± 3.5	27.7 ± 3.0	28.8 ± 3.9	28.4 ± 4.3	0.633
AML-average, mm	30.2 ± 3.6	30.2 ± 3.2	30.3 ± 3.9	29.1 ± 3.6	0.834
iAML-PLX, mm/m^2^	18.4 ± 2.7	18.4 ± 2.9	18.0 ± 2.9	17.5 ± 2.1	0.671
iAML-3CH, mm/m^2^	16.1 ± 2.4	15.7 ± 1.9	16.4 ± 2.4	16.8 ± 2.5	0.408
iAML-average, mm/m^2^	17.2 ± 2.3	17.0 ± 2.2	17.2 ± 2.4	17.1 ± 2.1	0.981

One patient with pathogenic variants in *GAA* was excluded. ^1^Pathogenic or likely pathogenic mutation or damaging mtDNA variant; ^§^p, *p*-value for ANOVA. *p < 0.05, **p < 0.01 versus no pathogenic or likely pathogenic variant group; ^†^*p* < 0.05 versus mitochondrial related variant group; See abbreviations in Table 1

### Measurement reproducibility of mitral leaflet length

With regard to intraobserver measurement variability, there was no difference between the first and second AML length measurements in PLX (29.8 ± 1.8 vs. 30.4 ± 2.1 mm, *P = 0.161*) or 3CH (27.8 ± 2.5 vs. 27.0 ± 2.6 mm, *P = 0.138*). Correlation between measurements was good for AML length in PLX (r = 0.835, *P = 0.003*) and 3CH (r = 0.827, *P = 0.003*).

## Discussion

In this study, we confirmed that mitral leaflet size was significantly larger in patients with HCM than in controls. Among these patients, significant and independent correlations were observed between mitral leaflet size and BSA, LV volume, LV mass by CMR, degree of MR and LAWS. An extensive HCM panel showed no independent relationships of genetic factors with mitral leaflet size.

### Chamber remodeling and hemodynamic load to mitral leaflet length

Previous studies have shown that hemodynamic load induces mitral leaflet development. In addition, adaptive mitral leaflet growth may be observed in adults [19]. Our results also support the hypothesis that LV end-systolic valve stress from the LV side and LA end-diastolic valve wall stress from the LA side correlate with AML size. We confirmed that leaflet lengths contribute to the development of a trans-LVOT pressure gradient. Our data showing a significant correlation between MR grade and mitral leaflet length suggest that hemodynamic load contributes to leaflet elongation. However, whether increase in the trans-LVOT pressure gradient and MR is a cause or result of AML elongation is unclear. There are principally two types of cells found in mitral leaflet tissue, namely, endothelial cells that cover the surface of the cusps and interstitial cells (ICs) that form a network within the extracellular matrix (ECM) in the body of the cusp [6]. Both cell types exhibit unique functions that are different from those of other endothelial cells and ICs found throughout the body. Valve ICs express a complex pattern of cell surface, cytoskeletal, and muscle proteins. These are able to bind to- and communicate with each other and the ECM. Although, endothelial cells on the outflow and inflow surfaces of the valve differ from one another, [6, 20] valve stresses on both sides would promote leaflet elongation even in overt HCM status as supported by our study results.

### Genetic contribution to mitral leaflet length

In our results, patients with HCM had larger iAMLs than age- and sex-matched controls, indicating that genetic factors contribute to mitral leaflet elongation. However, we could not find a direct relationship between leaflet size and sarcomere genes, phenocopy genes, mitochondria-related nDNA or mtDNA in the stage of overt HCM. Previous some studies showed that sarcomere gene mutations promote mitral leaflet elongation in subclinical HCM [2, 7], however after development of myocardial hypertrophy and LV remodeling, hemodynamic and geometrical factors predominantly affect mitral leaflet elongation. Therefore, mitral leaflet elongation could be a secondary phenotype following myocardial hypertrophy. These findings are supported by the previous study results of larger leaflets size in overt HCM patients than in subclinical HCM, in spite of same sarcomere gene mutations [2, 7].

Embryologically, the mitral leaflet originates from the atrio-ventricular annulus, which is adjacent to the basal portion of the left ventricle [20]. According to our study, leaflet size was smaller for pure ApHCM than for others, including asymmetrical basal septal hypertrophy, suggesting that hypertrophy signals affect adjacent AML growth through hemodynamic load or genetic predisposition to basal myocardial hypertrophy. Why the AML is larger in classical HCM than in ApHCM might be explained by genetic contribution, because of more prevalent in mitochondria-related gene or mtDNA mutations and less prevalent in sarcomere gene mutations in ApHCM. Troponin I and T genes, included in thin sarcomere protein gene, were shown to be related to valve IC activation [5]. Thin filament or regulatory genes were more related to mitral leaflet elongation in the present and a previous study [4], which suggests that mitral leaflet size is determined by multiple genes and modified by chronic hemodynamic load and chamber remodeling. However, this relationship was significantly attenuated after controlling for body size or LV size.

A recent study revealed that mutations in DCHS1 gene cause mitral valve prolapse with elongation [21], which suggests that MV specific gene might directly affect mitral leaflet enlargement even in HCM. Therefore, studies for revealing genetic factors not only sarcomere genes but also genes coding MV ICs need to be performed in HCM. This effort would open new therapeutic option for preventing obstructive HCM by genetic manipulation.

### Limitations

This study has several limitations. First, only AML size was measured with echocardiography. Although cardiovascular magnetic resonance (CMR) has been used good imaging modality for both AML and posterior mitral leaflet, a review of MV leaflet size showed that CMR cannot perfectly measure the distance. AML usually represents the main pathology in HCM, and leaflet length measurements were performed in two different views, with the average value used to reduce measurement errors. Second, our extensive HCM gene panel and mtDNA analysis did not assess rare variant contribution to mitral leaflet size. Third, the enrolled study population number was not large enough to reach statistical power. The cost of next-generation sequencing is continuously decreasing; hence, extensive gene panel analyses to evaluate multigenic contributions to leaflet size in a large HCM registry is warranted.

## Conclusion

Mitral leaflet elongation was a unique finding of HCM. However, the leaflet size was more related to LV geometry and hypertrophy pattern rather than genetic factors within overt HCM, suggesting that mitral leaflet elongation is due to gene–hemodynamic interaction and chamber remodeling.

## Supplementary information

**Additional file 1.** Method S1. DNA preparation; Method S2. Library construction and sequencing of the HCM gene panel; Method S3. Library construction and mtDNA sequencing; Method S4. Data analysis of the mitochondrial genome.

**Additional file 2. Table S1.** Summary of 82 genes associated with hypertrophic cardiomyopathy; **Table S2.** Likely pathogenic or Pathogenic variants in the 33 sarcomere associated genes classified according to the 2015 American College of Medical Genetics guidelines; **Table S3.** Likely pathogenic or Pathogenic variants in the 6 non-sarcomere genes and the 44 mitochondria-related nuclear genes.

## Data Availability

The datasets used and/or analysed during the current study are available from the corresponding author on reasonable request.

## Abbreviations

3CH: three-chamber view
AML: Anterior mitral leaflet
ApHCM: Apical hypertrophic cardiomyopathy
CMR: Cardiac magnetic resonance imaging
ECM: Extracellular matrix
HCM: Hypertrophic cardiomyopathy
IC: Interstitial cell
LA: Left atrial
LV: Left ventricular
LVESV: Left ventricular end-systolic volume
LVOT: Left ventricular outflow tract
MR: Mitral regurgitation
PLX: Parasternal long axis view
