# Supplementary material for: Genetic relevance and determinants of mitral leaflet size in hypertrophic cardiomyopathy
Source: Cardiovasc Ultrasound. 2019 Oct 28;17:21. doi: 10.1186/s12947-019-0171-1 (PMC6819388; doi:10.1186/s12947-019-0171-1)
Supplement: Supplementary file 1 — Additional file 1. Method S1. DNA preparation; Method S2. Library construction and sequencing of the HCM gene panel; Method S3. Library construction and mtDNA sequencing; Method S4. Data analysis of the mitochondrial genome. [file 12947_2019_171_MOESM1_ESM.docx]

**Method S1. DNA preparation**

Genomic DNA was extracted from EDTA-treated whole blood samples by using a QIAamp DNA Blood Mini kit (Qiagen, Hilden, Germany) on a QIAcube automatic nucleic acid extrac­tion instrument (Qiagen) according to the manufacturer’s in­structions. gDNA concentration and purity were assessed by a Nanodrop 1000 spectrometer (Thermo Scientific, Waltham, MA, USA). Mean gDNA yield and A_260/280_ values were 87.0 ng/μL and 1.8, respectively. The DNA samples were used to analyze mtDNA and the HCM gene panel (nDNA).

**Method S2. Library construction and sequencing of the HCM gene panel**

For targeted sequencing, DNA fragments were enriched by solution-based hybridization capture and sequenced on an Illumina Hiseq2500 platform (Illumina, San Diego, CA, USA) with the 2 × 150 base pair paired-end read module. Genomic DNA was sheared using an Adaptive Focused Acoustics™ with a Covaris Focused-ultrasonicator (Covaris, Inc., Woburn, MA, USA). The quality and quantity of sheared DNA were assessed using the Agilent 2200 Tape Station system with Agilent D1000 ScreenTape (Agilent Technologies, Santa Clara, CA, USA) according to the manufacturer’s instructions. Capture probes were customized and produced by Celemics, Inc. (Seoul, Korea) to cover coding sequence regions of 82 target genes. Purification and clean-up of samples were also performed with AMPure beads. NGS library amplification was performed using a KAPA Library Amplification Kit (Kapa Biosystems, Inc., Wilmington, MA, USA) according to the manufacturer’s instructions. Library preparation, hybridization, capture procedure, and sequencing were performed by Celemics according to the protocols recommended by the Celemics User Manual Ver 2.1 (<http://www.celemics.com/home/>).

**Method S3. Library construction and mtDNA sequencing**

The complete mtDNA was amplified by using four overlapping pairs of primers.(1) Library preparations were performed following the manufacturer’s instructions (Ion XpressPlus Fragment Library Kit; Thermo Fisher Scientific, Waltham, MA, USA) for 400 single-end reads. Library material was purified using AMPure beads (Beckman Coulter, Brea, CA, USA). For multiplexing of the samples, each DNA library was barcoded using different ligation adaptors. The fragmented and adaptor ligated libraries were selected following electrophoretic separation with the E-gel SizeSelect gel (Thermo Fisher Scientific) following the manufacturer’s recommendations. Subsequent emulsion polymerase chain reaction and enrichment of the sequencing beads of the pooled libraries was performed using the OneTouch system (Thermo Fisher Scientific) according to the manufacturer’s protocol. Finally, sequencing was conducted on the 318 chip using Ion PGM Hi-Q Sequencing Kit on the Ion Torrent Personal Genome Machine (Thermo Fisher Scientific).

**Method S4. Data analysis of the mitochondrial genome**

Quality-filtered sequences were aligned to the Revised Cambridge Reference Sequence (rCRS; NC_012920) and analyzed.(2) Basic data analysis was performed using Torrent Suite Software version 5.2.1 with the default parameters (Life Technologies, Carlsbad, CA, USA) and the plug-in VariantCaller for mtDNA employing TMAP Smith–Waterman alignment optimization.(3) The output of the variant caller was presented in a tabular format as a list of variations to the revised Cambridge Reference (rCRS) with total coverage and variant frequency values. Variant detection was called to detect insertions and deletions as well as single-nucleotide polymorphisms with reference to the rCRS. Visual inspection of the mapped data was performed using Integrated Genomics Viewer 2.3 software (IGV; Broad Institute, Cambridge, MA, USA). Mitochondrial genome databases, including MITOMAP(4) and Human Mitochondrial Genome Database (mtDB)(5) and Phylotree(6) were referred to validate the detected variants.

**References**

1. Wang HW, Jia X, Ji Y, Kong QP, Zhang Q, Yao YG, et al. Strikingly different penetrance of LHON in two Chinese families with primary mutation G11778A is independent of mtDNA haplogroup background and secondary mutation G13708A. Mutation research. 2008;643(1-2):48-53.

2. Andrews RM, Kubacka I, Chinnery PF, Lightowlers RN, Turnbull DM, Howell N. Reanalysis and revision of the Cambridge reference sequence for human mitochondrial DNA. Nature genetics. 1999;23(2):147.

3. Li H, Homer N. A survey of sequence alignment algorithms for next-generation sequencing. Briefings in bioinformatics. 2010;11(5):473-83.

4. Brandon MC, Lott MT, Nguyen KC, Spolim S, Navathe SB, Baldi P, et al. MITOMAP: a human mitochondrial genome database--2004 update. Nucleic acids research. 2005;33(Database issue):D611-3.

5. Ingman M, Gyllensten U. mtDB: Human Mitochondrial Genome Database, a resource for population genetics and medical sciences. Nucleic acids research. 2006;34(Database issue):D749-51.

6. Chaitanya L, Ralf A, van Oven M, Kupiec T, Chang J, Lagace R, et al. Simultaneous Whole Mitochondrial Genome Sequencing with Short Overlapping Amplicons Suitable for Degraded DNA Using the Ion Torrent Personal Genome Machine. Human mutation. 2015;36(12):1236-47.
