# Supplementary material for: Genetic relevance and determinants of mitral leaflet size in hypertrophic cardiomyopathy
Source: Cardiovasc Ultrasound. 2019 Oct 28;17:21. doi: 10.1186/s12947-019-0171-1 (PMC6819388; doi:10.1186/s12947-019-0171-1)
Supplement: Supplementary file 2 — Additional file 2. Table S1. Summary of 82 genes associated with hypertrophic cardiomyopathy; Table S2. Likely pathogenic or Pathogenic variants in the 33 sarcomere associated genes classified according to the 2015 American College of Medical Genetics guidelines; Table S3. Likely pathogenic or Pathogenic variants in the 6 non-sarcomere genes and the 44 mitochondria-related nuclear genes. [file 12947_2019_171_MOESM2_ESM.docx]

| **Table S1.** Summary of 82 genes associated with hypertrophic cardiomyopathy | | | | | | |
| --- | --- | --- | --- | --- | --- | --- |
| Category | Gene | OMIM number | Transcript reference | Phenotype | Inheritance | Reference |
| Sarcomere associated genes (validated) | ACTC1 | * 102540 | NM_005159.4 | Cardiomyopathy, hypertrophic, 11 | AD | (1) |
|  | MYBPC3 | * 600958 | NM_000256.3 | Cardiomyopathy, hypertrophic, 4 | AD | (1) |
|  | MYH7 | * 160760 | NM_000257.3 | Cardiomyopathy, hypertrophic, 1 | AD | (1) |
|  | MYL2 | * 160781 | NM_000432.3 | Cardiomyopathy, hypertrophic, 1 | AD | (1) |
|  | MYL3 | * 160790 | NM_000258.2 | Cardiomyopathy, hypertrophic, 8 | AD | (1) |
|  | TNNI3 | * 191044 | NM_000363.4 | Cardiomyopathy, hypertrophic, 7 | AD | (1) |
|  | TNNT2 | * 191045 | NM_001001430.2 | Cardiomyopathy, hypertrophic, 2 | AD | (1) |
|  | TPM1 | * 191010 | NM_001018005.1 | Cardiomyopathy, hypertrophic, 3 | AD | (1) |
| Sarcomere associated genes (putative) | CSRP3 | * 600824 | NM_003476.4 | Cardiomyopathy, hypertrophic, 12 | AD | (1) |
|  | FHL1 | * 300163 | NM_001449.4 | Emery-Dreifuss muscular dystrophy 6, X-linked | XLR | (1) |
|  | PLN | * 172405 | NM_002667.3 | Cardiomyopathy, hypertrophic, 18 | AD | (1) |
|  | ACTN2 | * 102573 | NM_001103.3 | Cardiomyopathy, hypertrophic, 23, with or without LVNC | AD | (1) |
|  | CRYAB | * 123590 | NM_001885.1 | Cardiomyopathy, dilated, 1II | AD | (1) |
|  | MYOZ2 | * 605602 | NM_016599.4 | Cardiomyopathy, hypertrophic, 16 | AD | (1) |
|  | MYH6 | * 160710 | NM_002471.3 | Cardiomyopathy, hypertrophic, 14 | AD | (1) |
|  | TNNC1 | * 191040 | NM_003280.2 | Cardiomyopathy, hypertrophic, 13 | AD | (1) |
|  | ANKRD1 | * 609599 | NM_014391.2 | Hypertrophic Cardiomyopathy | Not evaluated | (1) |
|  | CAV3 | * 601253 | NM_033337.2 | Cardiomyopathy, familial hypertrophic | AD | (1) |
|  | JPH2 | * 605267 | NM_020433.4 | Cardiomyopathy, hypertrophic, 17 | AD | (1) |
|  | LDB3 | * 605906 | NM_007078.2 | Cardiomyopathy, hypertrophic, 24 | AD | (1) |
|  | MYLK2 | * 606566 | NM_033118.3 | Cardiomyopathy, hypertrophic, 1, digenic | AD | (1) |
|  | MYOM1 | * 603508 | NM_003803.3 | Hypertrophic Cardiomyopathy | Not evaluated | (1) |
|  | MYPN | * 608517 | NM_032578.3 | Cardiomyopathy, hypertrophic, 22 | AD | (1) |
|  | NEXN | * 613121 | NM_144573.3 | Cardiomyopathy, hypertrophic, 2 | AD | (1) |
|  | TCAP | * 604488 | NM_003673.3 | Cardiomyopathy, hypertrophic, 25 | AD | (1) |
|  | BAG3 | * 603883 | NM_004281.3 | Cardiomyopathy, dilated, 1HH | AD | (2) |
|  | CASQ2 | * 114251 | NM_001232.3 | Ventricular tachycardia, catecholaminergic polymorphic, 2 | AR | (1) |
|  | MYO6 | * 600970 | NM_004999.3 | Deafness, autosomal dominant 22, with hypertrophic cardiomyopathy | AD | (1) |
|  | OBSCN | * 608616 | NM_052843.3 | Inherited cardiomyopathies | Not evaluated | (1) |
|  | RYR2 | * 180902 | NM_001035.2 | Arrhythmogenic right ventricular dysplasia 2 | AD | (3) |
|  | TNNC2 | * 191039 | NM_003279.2 | Cardiomyopathy | Not evaluated | (4) |
|  | TTN | * 188840 | NM_001267550.1 | Cardiomyopathy, familial hypertrophic, 9 | AD/AR | (5) |
|  | VCL | * 193065 | NM_014000.2 | Cardiomyopathy, hypertrophic, 15 | AD | (1) |
| Phenocopies genes | GAA | * 606800 | NM_001079803.2 | Pompe disease | AR | (6) |
|  | LAMP2 | * 309060 | NM_002294.2 | Danon disease | XLD | (6) |
|  | PRKAG2 | * 602743 | NM_016203.3 | Wolff-Parkinson-White syndrome, conduction disease | AD | (6) |
|  | PTPN11 | * 176876 | NM_002834.3 | LEOPARD syndrome, Noonan syndrome | AD | (6) |
|  | TTR | * 176300 | NM_000371.3 | Transthyretin amyloidosis | AD | (7) |
| Mitochondria- related nuclear DNA genes | AARS2 | * 612035 | NM_020745.3 | Combined oxidative phosphorylation deficiency 8 | AR | (8) |
|  | ACAD9 | * 611103 | NM_014049.4 | Mitochondrial complex I deficiency due to ACAD9 deficiency | AR | (9) |
|  | ACADVL | * 609575 | NM_000018.3 | VLCAD deficiency | AR | (10) |
|  | AGK | * 610345 | NM_018238.3 | Sengers Syndrome | AR | (11) |
|  | COA5 | * 613920 | NM_001008215.1 | Cardioencephalomyopathy, fatal infantile, due to cytochrome c oxidase deficiency 3 | AR | (12) |
|  | COA6 | * 614772 | NM_001206641.2 | Cardioencephalomyopathy, fatal infantile, due to cytochrome c oxidase deficiency 4 | AR | (13) |
|  | COQ2 | * 609825 | NM_015697.7 | Coenzyme Q10 deficiency, primary, 1 | AR | (14) |
|  | COQ4 | * 612898 | NM_016035.3 | Coenzyme Q10 deficiency, primary, 7 | AR | (14) |
|  | COQ9 | * 612837 | NM_020312.3 | Coenzyme Q10 deficiency, primary, 5 | AR | (14) |
|  | COX10 | * 602125 | NM_001303.3 | Leigh syndrome due to mitochondrial COX4 deficiency | AR | (15) |
|  | COX14 | * 614478 | NM_032901.2 | Mitochondrial complex IV deficiency | AR | (16) |
|  | COX15 | * 603646 | NM_004376.5 | Leigh syndrome due to cytochrome c oxidase deficiency | AR | (17) |
|  | COX6B1 | * 124089 | NM_001863.4 | Mitochondrial complex IV deficiency | AR | (18) |
|  | CPT2 | * 600650 | NM_000098.2 | CPT II deficiency | AR | (19) |
|  | ECHS1 | * 602292 | NM_004092.3 | Mitochondrial short-chain enoyl-CoA hydratase 1 deficiency | AR | (20) |
|  | ELAC2 | * 605367 | NM_018127.6 | Combined oxidative phosphorylation deficiency 17 | AR | (21) |
|  | FOXRED1 | * 613622 | NM_017547.3 | Leigh syndrome due to mitochondrial complex I deficiency | AR | (22) |
|  | GTPBP3 | * 608536 | NM_032620.3 | Combined oxidative phosphorylation deficiency 23 | AR | (23) |
|  | HADHB | * 143450 | NM_000183.2 | Trifunctional protein deficiency | AR | (24) |
|  | LRPPRC | * 607544 | NM_133259.3 | Leigh syndrome, French-Canadian type | AR | (25) |
|  | MRPL3 | * 607118 | NM_007208.3 | Combined oxidative phosphorylation deficiency 9 | AR | (26) |
|  | MRPL44 | * 611849 | NM_022915.3 | Combined oxidative phosphorylation deficiency 16 | AR | (27) |
|  | MRPS22 | * 605810 | NM_020191.2 | Combined oxidative phosphorylation deficiency 5 | AR | (17) |
|  | MTO1 | * 614667 | NM_012123.3 | Combined oxidative phosphorylation deficiency 10 | AR | (28) |
|  | NDUFA10 | * 603835 | NM_004544.3 | Leigh syndrome | AR | (29) |
|  | NDUFA11 | * 612638 | NM_175614.4 | Mitochondrial complex I deficiency | AR | (30) |
|  | NDUFA2 | * 602137 | NM_002488.4 | Leigh syndrome due to mitochondrial complex I deficiency | AR | (17) |
|  | NDUFAF1 | * 606934 | NM_016013.2 | Mitochondrial complex I deficiency | AR | (31) |
|  | NDUFS2 | * 602985 | NM_004550.4 | Mitochondrial complex I deficiency | AR | (32) |
|  | NDUFS4 | * 602694 | NM_002495.2 | Mitochondrial complex I deficiency | AR | (33) |
|  | NDUFS8 | * 602141 | NM_002496.3 | Leigh syndrome due to mitochondrial complex I deficiency | AR | (34) |
|  | NDUFV2 | * 600532 | NM_021074.4 | Mitochondrial complex I deficiency | AR | (17) |
|  | PCCB | * 232050 | NM_000532.4 | Propionicacidemia | AR | (35) |
|  | SCO2 | * 604272 | NM_005138.2 | Cardioencephalomyopathy, fatal infantile, due to cytochrome c oxidase deficiency 1 | AR | (17) |
|  | SDHD | * 602690 | NM_003002.2 | Mitochondrial complex II deficiency | AR | (36) |
|  | SLC22A5 | * 603377 | NM_003060.3 | Carnitine deficiency, systemic primary | AR | (37) |
|  | SLC25A20 | * 613698 | NM_000387.5 | Carnitine-acylcarnitine translocase deficiency | AR | (38) |
|  | SLC25A3 | * 600370 | NM_005888.3 | Mitochondrial phosphate carrier deficiency | Not evaluated | (17) |
|  | SLC25A4 | * 103220 | NM_001151.3 | Mitochondrial DNA depletion syndrome 12A /12B | AD/AR | (39) |
|  | SURF1 | * 185620 | NM_003172.2 | Leigh syndrome, due to COX IV deficiency | AR | (40) |
|  | TMEM70 | * 612418 | NM_017866.5 | Mitochondrial complex V (ATP synthase) deficiency, nuclear type 2 | AR | (17) |
|  | TRMT5 | * 611023 | NM_020810.3 | Combined oxidative phosphorylation deficiency 26 | AR | (41) |
|  | TSFM | * 604723 | NM_001172696.1 | Combined oxidative phosphorylation deficiency 3 | AR | (17) |
|  | YARS2 | * 610957 | NM_001040436.2 | Myopathy, lactic acidosis, and sideroblastic anemia 2 | AR | (42) |

AD: Autosomal dominant, AR: Autosomal recessive, XLR: X-linked recessive

**Table S2.** Likely pathogenic or Pathogenic variants in the 33 sarcomere associated genes classified according to the 2015 American College of Medical Genetics guidelines

| Case | Gene | Reference sequence | DNA Change | Amino Acid Change | Zygosity | Align GVGD^a^ | SIFT^b^ | Mutation Taster^b^ | Clinvar accession | Clinvar variant class | HGMD accession | HGMD phenotype | HGMD variant class | MAF (ExAC) | MAF (KRGDB) | dbSNP | OMGL/LMM^c^ |
| --- | --- | --- | --- | --- | --- | --- | --- | --- | --- | --- | --- | --- | --- | --- | --- | --- | --- |
| YMC-2 | *TNNI3* | NM_000363.4 | c.434G>A | p.Arg145Gln | Het | Class C0 (GV: 241.65 - GD: 0.00) | T (score: 0.06) | D (p-value: 0.957) | RCV000208273.1 / RCV000159223.4 | LP / P | CM971497 | HCM | DM | 0.00002490 | 0.000909 | rs397516349 | LP |
| YMC-3 | *MYH7* | NM_000257.3 | c.1324C>T | p.Arg442Cys | Het | Class C55 (GV: 26.00 - GD: 177.62) | D (score: 0) | D (p-value: 1) | RCV000162335.1 / RCV000464365.1 | LP / P | CM066924 | HCM | DM | 0.00000824 | 0.00000000 | rs148808089 | LP/ P |
| YMC-4 | *MYH7* | NM_000257.3 | c.1324C>T | p.Arg442Cys | Het | Class C55 (GV: 26.00 - GD: 177.62) | D (score: 0) | D (p-value: 1) | RCV000162335.1 / RCV000464365.1 | LP / P | CM066924 | HCM | DM | 0.00000824 | 0.00000000 | rs148808089 | LP/ P |
| YMC-7 | *MYBPC3* | NM_000256.3 | c.1484G>A | p.Arg495Gln | Het | Class C35 (GV: 0.00 - GD: 42.81) | D (score: 0) | D (p-value: 1) | RCV000168090.5 | LP / P | CM981324 | HCM | DM | 0.00000829 | 0.00000000 | rs200411226 | VUS/LP |
| YMC-15 | *TNNI3* | NM_000363.4 | c.434G>A | p.Arg145Gln | Het | Class C0 (GV: 241.65 - GD: 0.00) | T (score: 0.06) | D (p-value: 0.957) | RCV000208273.1 / RCV000159223.4 | LP / P | CM971497 | HCM | DM | 0.00002490 | 0.000909 | rs397516349 | LP |
| YMC-17 | *MYBPC3* | NM_000256.3 | c.2512G>T | p.Glu838* | Het | (-) | (-) | (-) | (-) | (-) | CM1313299 | HCM | DM | 0.00000000 | 0.00000000 | Novel | (-) |
| YMC-20 | *MYH7* | NM_000257.3 | c.4130C>T | p.Thr1377Met | Het | Class C15 (GV: 76.97 - GD: 81.04) | D (score: 0.03) | D (p-value: 1) | RCV000035886.3/RCV000208315.1 | VUS / LP | CM034558 | HCM | DM | 0.00000000 | 0.000455 | rs397516201 | VUS/LP |
| YMC-25 | *MYBPC3* | NM_000256.3 | c.1000G>A | p.Glu334Lys | Het | Class C55 (GV: 0.00 - GD: 56.87) | D (score: 0) | D (p-value: 1) | RCV000168764.2 | LP | CM073211 | HCM | DM | 0.00033786 | 0.006364 | rs573916965 | VUS favour P |
| YMC-27 | *TNNI3* | NM_000363.4 | c.434G>A | p.Arg145Gln | Het | Class C0 (GV: 241.65 - GD: 0.00) | T (score: 0.06) | D (p-value: 0.957) | RCV000208273.1 / RCV000159223.4 | LP / P | CM971497 | HCM | DM | 0.00002490 | 0.000909 | rs397516349 | LP |
| YMC-30 | *TNNI3* | NM_000363.4 | c.434G>A | p.Arg145Gln | Het | Class C0 (GV: 241.65 - GD: 0.00) | T (score: 0.06) | D (p-value: 0.957) | RCV000208273.1 / RCV000159223.4 | LP / P | CM971497 | HCM | DM | 0.00002490 | 0.000909 | rs397516349 | LP |
| YMC-32 | *MYBPC3* | NM_000256.3 | c.86delT | p.Phe29Serfs*10 | Het | (-) | (-) | (-) | (-) | (-) | (-) | (-) | (-) | 0.00000000 | 0.00000000 | Novel | (-) |
| YMC-37 | *MYBPC3* | NM_000256.3 | c.2459G>A | p.Arg820Gln | Het | Class C35 (GV: 0.00 - GD: 42.81) | D (score: 0) | D (p-value: 1) | RCV000158159.3/RCV000009148.6 | LP | CM034547 | HCM | DM | 0.00001657 | 0.00000000 | rs2856655 | VUS/LP |
|  | *MYH7* | NM_000257.3 | c.1324C>T | p.Arg442Cys | Het | Class C55 (GV: 26.00 - GD: 177.62) | D (score: 0) | D (p-value: 1) | RCV000162335.1 / RCV000464365.1 | LP / P | CM066924 | HCM | DM | 0.00000824 | 0.00000000 | rs148808089 | LP/ P |
| YMC-40 | *MYH7* | NM_000257.3 | c.4130C>T | p.Thr1377Met | Het | Class C15 (GV: 76.97 - GD: 81.04) | D (score: 0.03) | D (p-value: 1) | RCV000035886.3/RCV000208315.1 | VUS / LP | CM034558 | HCM | DM | 0.00000000 | 0.000455 | rs397516201 | VUS/LP |
| YMC-41 | *MYBPC3* | NM_000256.3 | c.2833_2834del | p.Arg945Glyfs | Het | (-) | (-) | (-) | RCV000035530.2 | P | CD022562 | HCM | DM | 0.00000000 | 0.00000000 | rs397515987 | (-) |
| YMC-43 | *MYBPC3* | NM_000256.3 | c.1505G>A | p.Arg502Gln | Het | Class C35 (GV: 0.00 - GD: 42.81) | D (score: 0) | D (p-value: 1) | RCV000168303.2 | P | CM981325 | HCM | DM | 0.00000000 | 0.00000000 | rs397515907 | P |
|  | *JPH2* | NM_020433.4 | c.1037_1047del | p.Leu346Hisfs*50 | Het | (-) | (-) | (-) | (-) | (-) | (-) | (-) | (-) | 0.00000000 | 0.00000000 | Novel | (-) |
| YMC-51 | *MYBPC3* | NM_000256.3 | c.2067+1G>A | (-) | Het | (-) | (-) | (-) | (-) | (-) | CS063340 | HCM | DM | 0.00000000 | 0.00160800 | (-) | (-) |
| YMC-52 | *MYH7* | NM_000257.3 | c.599C>T | p.Ala200Val | Het | Class C0 (GV: 353.86 - GD: 0.00) | D (score: 0.03) | D (p-value: 1) | (-) | (-) | (-) | (-) | (-) | 0.00000000 | 0.00000000 | Novel | (-) |
| YMC-54 | *TNNI3* | NM_000363.4 | c.485G>C | p.Arg162Pro | Het | Class C0 (GV: 243.06 - GD: 40.46) | D (score: 0.04) | D (p-value: 0.996 | RCV000036301.3 | LP | CM031380 | HCM | DM | 0.00000000 | 0.00000000 | rs397516354 | LP |
| YMC-55 | *MYBPC3* | NM_000256.3 | c.2272G>A | p.Gly758Ser | Het | Class C55 (GV: 0.00 - GD: 55.27) | D (score: 0) | D (p-value: 1) | (-) | (-) | (-) | (-) | (-) | 0.00000000 | 0.00000000 | Novel | (-) |
| YMC-60 | *MYBPC3* | NM_000256.3 | c.3805G>T | p.Glu1269* | Het | (-) | (-) | (-) | (-) | (-) | (-) | (-) | (-) | 0.00000000 | 0.00000000 | Novel | (-) |
|  | *MYBPC3* | NM_000256.3 | c.1000G>A | p.Glu334Lys | Het | Class C55 (GV: 0.00 - GD: 56.87) | D (score: 0) | D (p-value: 1) | RCV000168764.2 | LP | CM073211 | HCM | DM | 0.00033786 | 0.006364 | rs573916965 | VUS favour P |
| YMC-61 | *MYH7* | NM_000257.3 | c.2608C>T | p.Arg870Cys | Het | Class C55 (GV: 26.00 - GD: 177.62) | D (score: 0) | D (p-value: 1) | RCV000148707.1 / RCV000158554.1 | LP | CM003687 | HCM | DM | 0.00000824 | 0.000804 | rs138049878 | VUS |
| YMC-73 | *MYH7* | NM_000257.3 | c.1350G>T | p.Lys450Asn | Het | Class C0 (GV: 56.64 - GD: 42.43) | D (score: 0.01) | D (p-value: 1) | (-) | (-) | (-) | (-) | (-) | 0.00000000 | 0.00000000 | Novel | (-) |
| YMC-74 | *MYBPC3* | NM_000256.3 | c.2441_2443del | p.Lys814del | Het | (-) | (-) | (-) | RCV000157327.3 | VUS / LP | CD021840 | HCM | DM | 0.00000000 | 0.00000000 | rs727504288 | (-) |
| YMC-78 | *MYH7* | NM_000257.3 | c.4130C>T | p.Thr1377Met | Het | Class C15 (GV: 76.97 - GD: 81.04) | D (score: 0.03) | D (p-value: 1) | RCV000035886.3/RCV000208315.1 | VUS / LP | CM034558 | HCM | DM | 0.00000000 | 0.000455 | rs397516201 | VUS/LP |
| YMC-79 | *TNNC1* | NM_003280.2 | c.23C>T | p.Ala8Val | Het | Class C0 (GV: 353.86 - GD: 0.00) | T (score: 1) | D (p-value: 1) | RCV000037762.3 / RCV000159204.2 / RCV000013256.23 | VUS / LP / P | CM083571 | HCM | DM | 0.00000000 | 0.00000000 | rs267607125 | (-) |
| YMC-80 | *MYH7* | NM_000257.3 | c.2606G>C | p.Arg869Pro | Het | Class C0 (GV: 128.11 - GD: 0.00) | T (score: 0.25) | D (p-value: 1) | (-) | (-) | (-) | (-) | (-) | 0.00000000 | 0.00000000 | Novel | (-) |
| YMC-83 | *TNNI3* | NM_000363.4 | c.433C>G | p.Arg145Gly | Het | Class C0 (GV: 241.65 - GD: 24.28) | D (score: 0.02) | D (p-value: 0.961) | RCV000441050.1 / RCV000013231.25 | P | CM971498 | HCM | DM | 0.00000000 | 0.00000000 | rs104894724 | LP/ P |
| YMC-85 | *MYBPC3* | NM_000256.3 | c.2067+1G>A | (-) | Het | (-) | (-) | (-) | (-) | (-) | CS063340 | HCM | DM | 0.00000000 | 0.00160800 | (-) | (-) |
| YMC-88 | *MYBPC3* | NM_000256.3 | c.3490+1G>A | (-) | Het | (-) | (-) | (-) | RCV000009137.5 | P | CS971817 | HCM | DM | 0.00000000 | 0.00000000 | rs397516020 | P |
| YMC-89 | *MYBPC3* | NM_000256.3 | c.178G>T | p.Glu60* | Het | (-) | (-) | (-) | (-) | (-) | (-) | (-) | (-) | 0.00000000 | 0.00000000 | Novel | (-) |
| YMC-90 | *MYBPC3* | NM_000256.3 | c.2833_2834del | p.Arg945Glyfs | Het | (-) | (-) | (-) | RCV000035530.2 | P | CD022562 | HCM | DM | 0.00000000 | 0.00000000 | rs397515987 | (-) |
| YMC-93 | *MYH7* | NM_000257.3 | c.4130C>T | p.Thr1377Met | Het | Class C15 (GV: 76.97 - GD: 81.04) | D (score: 0.03) | D (p-value: 1) | RCV000035886.3/RCV000208315.1 | VUS / LP | CM034558 | HCM | DM | 0.00000000 | 0.000455 | rs397516201 | VUS/LP |
| YMC-94 | *TNNI3* | NM_000363.4 | c.434G>A | p.Arg145Gln | Het | Class C0 (GV: 241.65 - GD: 0.00) | T (score: 0.06) | D (p-value: 0.957) | RCV000208273.1 / RCV000159223.4 | LP / P | CM971497 | HCM | DM | 0.00002490 | 0.000909 | rs397516349 | LP |
| YMC-95 | *TNNI3* | NM_000363.4 | c.434G>A | p.Arg145Gln | Het | Class C0 (GV: 241.65 - GD: 0.00) | T (score: 0.06) | D (p-value: 0.957) | RCV000208273.1 / RCV000159223.4 | LP / P | CM971497 | HCM | DM | 0.00002490 | 0.000909 | rs397516349 | LP |
| YMC-96 | *MYBPC3* | NM_000256.3 | c.2441_2443del | p.Lys814del | Het | (-) | (-) | (-) | RCV000157327.3 | VUS / LP | CD021840 | HCM | DM | 0.00000000 | 0.00000000 | rs727504288 | (-) |
| YMC-104 | *MYBPC3* | NM_000256.3 | c.3763delG | p.Ala1255Profs*76 | Het | (-) | (-) | (-) | RCV000168826.2 | LP | (-) | (-) | (-) | 0.00000000 | 0.00000000 | rs786204362 | (-) |
| YMC-108 | *TNNI3* | NM_000363.4 | c.434G>A | p.Arg145Gln | Het | Class C0 (GV: 241.65 - GD: 0.00) | T (score: 0.06) | D (p-value: 0.957) | RCV000208273.1 / RCV000159223.4 | LP / P | CM971497 | HCM | DM | 0.00002490 | 0.000909 | rs397516349 | LP |
| YMC-110 | *MYH6* | NM_002471.3 | c.679dupG | p.Ala227Glyfs*24 | Het | (-) | (-) | (-) | (-) | (-) | (-) | (-) | (-) | 0.00000000 | 0.00000000 | Novel | (-) |
| YMC-114 | *MYBPC3* | NM_000256.3 | c.1000G>A | p.Glu334Lys | Het | Class C55 (GV: 0.00 - GD: 56.87) | D (score: 0) | D (p-value: 1) | RCV000168764.2 | LP | CM073211 | HCM | DM | 0.00033786 | 0.006364 | rs573916965 | VUS favour P |
|  | *MYH7* | NM_000257.3 | c.1324C>T | p.Arg442Cys | Het | Class C55 (GV: 26.00 - GD: 177.62) | D (score: 0) | D (p-value: 1) | RCV000162335.1 / RCV000464365.1 | LP / P | CM066924 | HCM | DM | 0.00000824 | 0.00000000 | rs148808089 | LP/ P |
| YMC-117 | *TNNI3* | NM_000363.4 | c.434G>A | p.Arg145Gln | Het | Class C0 (GV: 241.65 - GD: 0.00) | T (score: 0.06) | D (p-value: 0.957) | RCV000208273.1 / RCV000159223.4 | LP / P | CM971497 | HCM | DM | 0.00002490 | 0.000909 | rs397516349 | LP |
| YMC-121 | *MYBPC3* | NM_000256.3 | c.1000G>A | p.Glu334Lys | Hom | Class C55 (GV: 0.00 - GD: 56.87) | D (score: 0) | D (p-value: 1) | RCV000168764.2 | LP | CM073211 | HCM | DM | 0.00033786 | 0.006364 | rs573916965 | VUS favour P |
| YMC-123 | *TNNI3* | NM_000363.4 | c.434G>A | p.Arg145Gln | Het | Class C0 (GV: 241.65 - GD: 0.00) | T (score: 0.06) | D (p-value: 0.957) | RCV000208273.1 / RCV000159223.4 | LP / P | CM971497 | HCM | DM | 0.00002490 | 0.000909 | rs397516349 | LP |
| YMC-126 | *MYBPC3* | NM_000256.3 | c.1000G>A | p.Glu334Lys | Het | Class C55 (GV: 0.00 - GD: 56.87) | D (score: 0) | D (p-value: 1) | RCV000168764.2 | LP | CM073211 | HCM | DM | 0.00033786 | 0.006364 | rs573916965 | VUS favour P |
| YMC-127 | *MYBPC3* | NM_000256.3 | c.1000G>A | p.Glu334Lys | Het | Class C55 (GV: 0.00 - GD: 56.87) | D (score: 0) | D (p-value: 1) | RCV000168764.2 | LP | CM073211 | HCM | DM | 0.00033786 | 0.006364 | rs573916965 | VUS favour P |
| YMC-133 | *MYBPC3* | NM_000256.3 | c.2833_2834del | p.Arg945Glyfs | Het | (-) | (-) | (-) | RCV000035530.2 | P | CD022562 | HCM | DM | 0.00000000 | 0.00000000 | rs397515987 | (-) |
| YMC-134 | *MYBPC3* | NM_000256.3 | c.2067+1G>A | (-) | Het | (-) | (-) | (-) | (-) | (-) | CS063340 | HCM | DM | 0.00000000 | 0.00160800 | (-) | (-) |
| YMC-136 | *MYBPC3* | NM_000256.3 | c.2459G>A | p.Arg820Gln | Het | Class C35 (GV: 0.00 - GD: 42.81) | D (score: 0) | D (p-value: 1) | RCV000158159.3/RCV000009148.6 | LP | CM034547 | HCM | DM | 0.00001657 | 0.00000000 | rs2856655 | VUS/LP |
| YMC-137 | *TNNI3* | NM_000363.4 | c.433C>G | p.Arg145Gly | Het | Class C0 (GV: 241.65 - GD: 24.28) | D (score: 0.02) | D (p-value: 0.961) | RCV000441050.1 / RCV000013231.25 | P | CM971498 | HCM | DM | 0.00000000 | 0.00000000 | rs104894724 | LP/ P |
| YMC-141 | *MYL3* | NM_000258.2 | c.170C>G | p.Ala57Gly | Het | Class C0 (GV: 65.28 - GD: 60.00) | D (score: 0.02) | D (p-value: 1) | RCV000024471.3 / RCV000229595.2 | P | CM014210 | HCM | DM | 0.00000000 | 0.00160800 | rs139794067 | (-) |
| YMC-144 | *MYBPC3* | NM_000256.3 | c.1090+1G>A | (-) | Het | (-) | (-) | (-) | RCV000382204.1/RCV000211795.1 | LP / P | CS068101 | HCM | DM | 0.00000000 | 0.00000000 | rs727504269 | P |
| YMC-148 | *MYH6* | NM_002471.3 | c.2384G>A | p.Arg795Gln | Het | Class C35 (GV: 0.00 - GD: 42.81) | D (score: 0) | D (p-value: 1) | RCV000037469.3/RCV000171836.1/RCV000015210.21 | VUS / LP / P | CM020158 | HCM | DM | 0.00003298 | 0.000804 | rs267606907 | (-) |
| YMC-150 | TNNI3 | NM_000363.4 | c.434G>A | p.Arg145Gln | Het | Class C0 (GV: 241.65 - GD: 0.00) | T (score: 0.06) | D (p-value: 0.957) | RCV000159223.3\|RCV000200141.2\|RCV000208273.1 | LP / P | CM971497 | HCM | DM | 0.0000249 | 0.000909 | rs397516349 | LP |
| YMC-151 | MYBPC3 | NM_000256.3 | c.2067+1G>A | (-) | Het | (-) | (-) | (-) | RCV000521997.1 | P | CS063340 | HCM | DM | 0.00000000 | 0.00160772 | (-) | (-) |
| YMC-154 | MYH7 | NM_000257.3 | c.1988G>A | p.Arg663His | Het | Class C0 (GV: 241.74 - GD: 0.00) | T (score: 0.27) | D (p-value: 1) | RCV000035758.9 | P | CM993620 | HCM | DM | 0.00000000 | 0.00000000 | rs371898076 | (-) |
| YMC-159 | MYH7 | NM_000257.3 | c.1426C>T | p.Leu476Phe | Het | Class C15 (GV: 0.00 - GD: 21.82) | D (score: 0) | D (p-value: 1) | (-) | (-) | (-) | (-) | (-) | 0.00000000 | 0.00000000 | Novel | (-) |
| YMC-164 | MYBPC3 | NM_000256.3 | c.2458C>T | p.Arg820Trp | Het | Class C65 (GV: 0.00 - GD: 101.29) | D (score: 0) | D (p-value: 1) | RCV000176522.1 | LP | CM103890 | Cardiomyopathy | DM | 0.00000829 | 0.00000000 | rs775404728 | (-) |
| YMC-174 | MYBPC3 | NM_000256.3 | c.1000G>A | p.Glu334Lys | Het | Class C55 (GV: 0.00 - GD: 56.87) | D (score: 0) | D (p-value: 1 | RCV000168764.2 | LP | CM073211 | HCM | DM | 0.00033786 | 0.006364 | rs573916965 | VUS favour P |
| YMC-175 | MYH7 | NM_000257.3 | c.746G>A | p.Arg249Gln | Het | Class C0 (GV: 353.86 - GD: 0.00) | D (score: 0) | D (p-value: 1) | RCV000158761.2/RCV000229956.1 | P | CM910268 | HCM | DM | 0.00000000 | 0.00000000 | rs3218713 | P |
| YMC-177 | MYH7 | NM_000257.3 | c.4123T>C | p.Tyr1375His | Het | Class C65 (GV: 0.00 - GD: 83.33) | D (score: 0) | D (p-value: 1) | RCV000158634.1 | LP | CM167475 | HCM | DM | 0.00000000 | 0.00000000 | rs730880790 | (-) |
| YMC-180 | TNNI3 | NM_000363.4 | c.434G>A | p.Arg145Gln | Het | Class C0 (GV: 241.65 - GD: 0.00) | T (score: 0.06) | D (p-value: 0.957) | RCV000200141.2/RCV000159223.3 | P | CM971497 | HCM | DM | 0.0000249 | 0.000909 | rs397516349 | LP |
| YMC-189 | MYBPC3 | NM_000256.3 | c.3313_3314insGG | p.Ala1105Glyfs*85 | Het | (-) | (-) | (-) | (-) | (-) | (-) | (-) | (-) | 0.00000000 | 0.00000000 | Novel | (-) |
| YMC-192 | MYBPC3 | NM_000256.3 | c.3034C>T | p.Gln1012* | Het | (-) | (-) | (-) | RCV000158209.2 | P | CM034550 | HCM | DM | 0.00000000 | 0.00000000 | Novel | (-) |
| YMC-198 | MYH7 | NM_000257.3 | c.4066G>A | p.Glu1356Lys | Het | Class C15 (GV: 44.60 - GD: 56.87) | D (score: 0.04) | D (p-value: 1) | RCV000168900.1 | LP | CM042422 | HCM | DM | 0.00000000 | 0.00000000 | rs727503246 | P |
| YMC-205 | MYBPC3 | NM_000256.3 | c.2833_2834delCG | p.Arg945Glyfs*105 | Het | (-) | (-) | (-) | RCV000035530.2/RCV000158380.2 | P | CD022562 | HCM | DM | 0.00000000 | 0.00000000 | rs397515987 | P |
| YMC-206 | MYBPC3 | NM_000256.3 | c.2459G>A | p.Arg820Gln | Het | Class C35 (GV: 0.00 - GD: 42.81) | D (score: 0) | D (p-value: 1) | RCV000158159.3/RCV000009148.6 | LP | CM034547 | HCM | DM | 0.0000166 | 0.00000000 | rs2856655 | VUS/LP |
| YMC-207 | MYH7 | NM_000257.3 | c.1615A>G | p.Met539Val | Het | Class C0 (GV: 10.12 - GD: 20.52) | T (score: 0.05) | D (p-value: 1) | RCV000158890.1/RCV000205072.1 | LP/P | CM1516537 | HCM | DM | 0.00000000 | 0.00000000 | rs730880930 | (-) |
| YMC-214 | MYH7 | NM_000257.3 | c.4130C>T | p.Thr1377Met | Het | Class C15 (GV: 76.97 - GD: 81.04) | D (score: 0.03) | D (p-value: 1) | RCV000552931.1/RCV000518840.1/RCV000208315.2/RCV000617360.1 | VUS/LP | CM034558 | HCM | DM | 0.00000000 | 0.00000000 | rs397516201 | VUS favour P |

Abbreviations: ACMG, American College of Medical Genetics and Genomics; dbSNP, database of single nucleotide polymorphism; D, D or D; DCM, dilated cardiomyopathy; DM, disease-causing mutation; ExAC, Exome Aggregation Consortium; HCM, hypertrophic cardiomyopathy; Het, Heterozygous; Hom, Homozygous; HGMD, human gene mutation database; KRGDB, Korean Reference Genome Database; LP, Likely pathogenic; MAF, minor allele frequency; NM number, National Center for Biotechnology Information (NCBI) reference sequence;OMGL/LMM, the Oxford Molecular Genetics Laboratory and the Laboratory of Molecular Medicine; P, pathogenic; T, Tolerated; VUS, variant of uncertain significance. ^a^Align GVGD (http://agvgd.iarc.fr/) was used as a nucleotide-conservation prediction algorithm. ^b^ In silico tools including SIFT (http://sift.jcvi.org) and MutationTaster (http://www.mutationtaster.org) were used to assess the predicted impact of missense change ^c^Atlas of Cardiac Genetic Variation (https://cardiodb.org/ACGV/) used to assess the status of individual variants

**Table S3.** Likely pathogenic or Pathogenic variants in the 6 non-sarcomere genes and the 44 mitochondria-related nuclear genes

| Case | GENE | Reference sequence | DNA Change | Amino Acid Change | Align GVGD^a^ | SIFT^b^ | Mutation Taster^b^ | Clinvar accession | Clinvar phenotype | HGMD accession | HGMD phenotype | HGMD variant class | MAF (ExAC) | MAF (KRGDB) | dbSNP |
| --- | --- | --- | --- | --- | --- | --- | --- | --- | --- | --- | --- | --- | --- | --- | --- |
| YMC-12 | *AARS2* | NM_020745.3 | c.452T>C | p.Met151Thr | Class C0 (GV: 268.85 - GD: 38.84) | Tolerated (score: 0.07) | Disease causing (p-value: 1) | (-) | (-) | CM173156 | Ovarioleukodystrophy | DM | (-) | (-) | (-) |
|  | *ACADVL* | NM_000018.3 | c.865G>A | p.Gly289Arg | Class C0 (GV: 97.85 - GD: 42.00) | Deleterious (score: 0.01) | Disease causing (p-value: 1) | RCV000408960.1 | Very long chain acyl-CoA dehydrogenase deficiency | CM034380 | Very long chain acyl-CoA dehydrogenase deficiency | DM | 0.00009128 | 0.00045500 | rs200788251 |
| YMC-14 | *PCCB* | NM_000532.4 | c.1304A>G | p.Tyr435Cys | Class C55 (GV: 21.61 - GD: 191.71) | Deleterious (score: 0) | Disease causing (p-value: 1) | RCV000012798.24 | Propionic acidaemia | CM022051 | Propionic acidaemia | DM | 0.00004952 | 0.00318200 | rs121964961 |
| YMC-21 | *PCCB* | NM_000532.4 | c.1304A>G | p.Tyr435Cys | Class C55 (GV: 21.61 - GD: 191.71) | Deleterious (score: 0) | Disease causing (p-value: 1) | RCV000012798.24 | Propionic acidaemia | CM022051 | Propionic acidaemia | DM | 0.00004952 | 0.00318200 | rs121964961 |
| YMC-26 | *AGK* | NM_018238.3 | c.73G>T | p.Gly25* | (-) | (-) | (-) | (-) | (-) | (-) | (-) | (-) | (-) | (-) | (-) |
| YMC-38 | *GAA* | NM_001079803.2 | c.2171C>A | p.Ala724Asp | Class C0 (GV: 235.10 - GD: 79.30) | Deleterious (score: 0.04) | Disease causing (p-value: 1 | (-) | (-) | CM124040 | Glycogen storage disease 2 | DM | (-) | (-) | (-) |
| YMC-40 | *LRPPRC* | NM_133259.3 | c.4078G>A | p.Ala1360Thr | Class C0 (GV: 105.57 - GD: 26.10) | Deleterious (score: 0.04 | Disease causing (p-value: 1) | (-) | (-) | CM1715653 | Leigh syndrome | DM | 0.00078820 | 0.00884200 | rs147302249 |
| YMC-46 | *GTPBP3* | NM_032620.3 | c.8G>T | p.Arg3Leu | Class C0 (GV: 353.86 - GD: 0.00) | Tolerated (score: 0.08) | Disease causing (p-value: 0.74) | (-) | (-) | CM1413836 | Leigh syndrome | (-) | (-) | 0.00136400 | (-) |
| YMC-56 | *LRPPRC* | NM_133259.3 | c.3430C>T | p.Arg1144Cys | Class C0 (GV: 260.17 - GD: 0.00) | Tolerated (score: 0.15) | Disease causing (p-value: 1) | RCV000376700 | Leigh Syndrome | CM1715652 | Leigh syndrome | DM | 0.00014220 | (-) | rs760016065 |
| YMC-75 | *PCCB* | NM_000532.4 | c.1304A>G | p.Tyr435Cys | Class C55 (GV: 21.61 - GD: 191.71) | Deleterious (score: 0) | Disease causing (p-value: 1) | RCV000012798.24 | Propionic acidaemia | CM022051 | Propionic acidaemia | DM | 0.00004952 | 0.00318200 | rs121964961 |
| YMC-84 | *PCCB* | NM_000532.4 | c.1304A>G | p.Tyr435Cys | Class C55 (GV: 21.61 - GD: 191.71) | Deleterious (score: 0) | Disease causing (p-value: 1) | RCV000012798.24 | Propionic acidaemia | CM022051 | Propionic acidaemia | DM | 0.00004952 | 0.00318200 | rs121964961 |
| YMC-119 | *LRPPRC* | NM_133259.3 | c.4078G>A | p.Ala1360Thr | Class C0 (GV: 105.57 - GD: 26.10) | Deleterious (score: 0.04 | Disease causing (p-value: 1) | (-) | (-) | CM1715653 | Leigh syndrome | DM | 0.00078820 | 0.00884200 | rs147302249 |
| YMC-138 | *ACAD9* | NM_014049.4 | c.1552C>T | p.Arg518Cys | Class C0 (GV: 241.31 - GD: 79.30 | Deleterious (score: 0) | Disease causing (p-value: 1) | (-) | (-) | CM153909 | Acyl-Coenzyme dehydrogenase 9 deficiency | DM | 0.00010720 | (-) | rs150283105 |
| YMC-149 | *LRPPRC* | NM_133259.3 | c.4078G>A | p.Ala1360Thr | Class C0 (GV: 105.57 - GD: 26.10) | Deleterious (score: 0.04 | Disease causing (p-value: 1) | (-) | (-) | CM1715653 | Leigh syndrome | DM | 0.00078820 | 0.00884200 | rs147302249 |
| YMC-193 | *AARS2* | NM_020745.3 | c.609delT | p.Phe203Leufs*36 | (-) | (-) | (-) | (-) | (-) | (-) | (-) | (-) | 0.00000830 | (-) | (-) |
| YMC-194 | *PCCB* | NM_000532.4 | c.1304A>G | p.Tyr435Cys | Class C55 (GV: 21.61 - GD: 191.71) | Deleterious (score: 0) | Disease causing (p-value: 1) | RCV000012798.24 | Propionic acidaemia | CM022051 | Propionic acidaemia | DM | 0.00004952 | 0.00318200 | rs121964961 |
| YMC-196 | *PCCB* | NM_000532.4 | c.1304A>G | p.Tyr435Cys | Class C55 (GV: 21.61 - GD: 191.71) | Deleterious (score: 0) | Disease causing (p-value: 1) | RCV000012798.24 | Propionic acidaemia | CM022051 | Propionic acidaemia | DM | 0.00004952 | 0.00318200 | rs121964961 |

Abbreviations: ACMG, American College of Medical Genetics and Genomics; DCM, dilated cardiomyopathy; DM, disease-causing mutation; ExAC, Exome Aggregation Consortium; HCM, hypertrophic cardiomyopathy; HGMD, human gene mutation database; KRGDB, Korean Reference Genome Database; MAF, minor allele frequency; NM number, National Center for Biotechnology Information (NCBI) reference sequence; dbSNP, database of single nucleotide polymorphism; VUS, variant of uncertain significance

^a^Align GVGD (http://agvgd.iarc.fr/) was used as a nucleotide-conservation prediction algorithm.

^b^ In silico tools including SIFT (http://sift.jcvi.org) and MutationTaster (http://www.mutationtaster.org) were used to assess the predicted impact of missense change

**Reference**

1. Walsh R, Buchan R, Wilk A, John S, Felkin LE, Thomson KL, et al. Defining the genetic architecture of hypertrophic cardiomyopathy: re-evaluating the role of non-sarcomeric genes. European heart journal. 2017;38(46):3461-8.

2. Lee HC, Cherk SW, Chan SK, Wong S, Tong TW, Ho WS, et al. BAG3-related myofibrillar myopathy in a Chinese family. Clinical genetics. 2012;81(4):394-8.

3. Landstrom AP, Ackerman MJ. Beyond the cardiac myofilament: hypertrophic cardiomyopathy- associated mutations in genes that encode calcium-handling proteins. Current molecular medicine. 2012;12(5):507-18.

4. Li MX, Hwang PM. Structure and function of cardiac troponin C (TNNC1): Implications for heart failure, cardiomyopathies, and troponin modulating drugs. Gene. 2015;571(2):153-66.

5. Gigli M, Begay RL, Morea G, Graw SL, Sinagra G, Taylor MR, et al. A Review of the Giant Protein Titin in Clinical Molecular Diagnostics of Cardiomyopathies. Frontiers in cardiovascular medicine. 2016;3:21.

6. Yingchoncharoen T, Tang WW. Recent advances in hypertrophic cardiomyopathy. F1000prime reports. 2014;6:12.

7. Vermeer AMC, Janssen A, Boorsma PC, Mannens M, Wilde AAM, Christiaans I. Transthyretin amyloidosis: a phenocopy of hypertrophic cardiomyopathy. Amyloid : the international journal of experimental and clinical investigation : the official journal of the International Society of Amyloidosis. 2017;24(2):87-91.

8. Gotz A, Tyynismaa H, Euro L, Ellonen P, Hyotylainen T, Ojala T, et al. Exome sequencing identifies mitochondrial alanyl-tRNA synthetase mutations in infantile mitochondrial cardiomyopathy. American journal of human genetics. 2011;88(5):635-42.

9. Haack TB, Danhauser K, Haberberger B, Hoser J, Strecker V, Boehm D, et al. Exome sequencing identifies ACAD9 mutations as a cause of complex I deficiency. Nature genetics. 2010;42(12):1131-4.

10. Mathur A, Sims HF, Gopalakrishnan D, Gibson B, Rinaldo P, Vockley J, et al. Molecular heterogeneity in very-long-chain acyl-CoA dehydrogenase deficiency causing pediatric cardiomyopathy and sudden death. Circulation. 1999;99(10):1337-43.

11. Mayr JA, Haack TB, Graf E, Zimmermann FA, Wieland T, Haberberger B, et al. Lack of the mitochondrial protein acylglycerol kinase causes Sengers syndrome. American journal of human genetics. 2012;90(2):314-20.

12. Papadopoulou LC, Sue CM, Davidson MM, Tanji K, Nishino I, Sadlock JE, et al. Fatal infantile cardioencephalomyopathy with COX deficiency and mutations in SCO2, a COX assembly gene. Nature genetics. 1999;23(3):333-7.

13. Baertling F, M AMvdB, Hertecant JL, Al-Shamsi A, L PvdH, Distelmaier F, et al. Mutations in COA6 cause cytochrome c oxidase deficiency and neonatal hypertrophic cardiomyopathy. Human mutation. 2015;36(1):34-8.

14. Sondheimer N, Hewson S, Cameron JM, Somers GR, Broadbent JD, Ziosi M, et al. Novel recessive mutations in COQ4 cause severe infantile cardiomyopathy and encephalopathy associated with CoQ10 deficiency. Molecular genetics and metabolism reports. 2017;12:23-7.

15. Antonicka H, Leary SC, Guercin GH, Agar JN, Horvath R, Kennaway NG, et al. Mutations in COX10 result in a defect in mitochondrial heme A biosynthesis and account for multiple, early-onset clinical phenotypes associated with isolated COX deficiency. Human molecular genetics. 2003;12(20):2693-702.

16. Bourens M, Barrientos A. A CMC1-knockout reveals translation-independent control of human mitochondrial complex IV biogenesis. EMBO reports. 2017;18(3):477-94.

17. Schiff M, Ogier de Baulny H, Lombes A. Neonatal cardiomyopathies and metabolic crises due to oxidative phosphorylation defects. Seminars in fetal & neonatal medicine. 2011;16(4):216-21.

18. Abdulhag UN, Soiferman D, Schueler-Furman O, Miller C, Shaag A, Elpeleg O, et al. Mitochondrial complex IV deficiency, caused by mutated COX6B1, is associated with encephalomyopathy, hydrocephalus and cardiomyopathy. European journal of human genetics : EJHG. 2015;23(2):159-64.

19. Isackson PJ, Bennett MJ, Lichter-Konecki U, Willis M, Nyhan WL, Sutton VR, et al. CPT2 gene mutations resulting in lethal neonatal or severe infantile carnitine palmitoyltransferase II deficiency. Molecular genetics and metabolism. 2008;94(4):422-7.

20. Haack TB, Jackson CB, Murayama K, Kremer LS, Schaller A, Kotzaeridou U, et al. Deficiency of ECHS1 causes mitochondrial encephalopathy with cardiac involvement. Annals of clinical and translational neurology. 2015;2(5):492-509.

21. Haack TB, Kopajtich R, Freisinger P, Wieland T, Rorbach J, Nicholls TJ, et al. ELAC2 mutations cause a mitochondrial RNA processing defect associated with hypertrophic cardiomyopathy. American journal of human genetics. 2013;93(2):211-23.

22. Fassone E, Duncan AJ, Taanman JW, Pagnamenta AT, Sadowski MI, Holand T, et al. FOXRED1, encoding an FAD-dependent oxidoreductase complex-I-specific molecular chaperone, is mutated in infantile-onset mitochondrial encephalopathy. Human molecular genetics. 2010;19(24):4837-47.

23. Kopajtich R, Nicholls TJ, Rorbach J, Metodiev MD, Freisinger P, Mandel H, et al. Mutations in GTPBP3 cause a mitochondrial translation defect associated with hypertrophic cardiomyopathy, lactic acidosis, and encephalopathy. American journal of human genetics. 2014;95(6):708-20.

24. Orii KE, Aoyama T, Wakui K, Fukushima Y, Miyajima H, Yamaguchi S, et al. Genomic and mutational analysis of the mitochondrial trifunctional protein beta-subunit (HADHB) gene in patients with trifunctional protein deficiency. Human molecular genetics. 1997;6(8):1215-24.

25. Olahova M, Hardy SA, Hall J, Yarham JW, Haack TB, Wilson WC, et al. LRPPRC mutations cause early-onset multisystem mitochondrial disease outside of the French-Canadian population. Brain : a journal of neurology. 2015;138(Pt 12):3503-19.

26. Galmiche L, Serre V, Beinat M, Assouline Z, Lebre AS, Chretien D, et al. Exome sequencing identifies MRPL3 mutation in mitochondrial cardiomyopathy. Human mutation. 2011;32(11):1225-31.

27. Saada A, Shaag A, Arnon S, Dolfin T, Miller C, Fuchs-Telem D, et al. Antenatal mitochondrial disease caused by mitochondrial ribosomal protein (MRPS22) mutation. Journal of medical genetics. 2007;44(12):784-6.

28. Ghezzi D, Baruffini E, Haack TB, Invernizzi F, Melchionda L, Dallabona C, et al. Mutations of the mitochondrial-tRNA modifier MTO1 cause hypertrophic cardiomyopathy and lactic acidosis. American journal of human genetics. 2012;90(6):1079-87.

29. Hoefs SJ, van Spronsen FJ, Lenssen EW, Nijtmans LG, Rodenburg RJ, Smeitink JA, et al. NDUFA10 mutations cause complex I deficiency in a patient with Leigh disease. European journal of human genetics : EJHG. 2011;19(3):270-4.

30. Berger I, Hershkovitz E, Shaag A, Edvardson S, Saada A, Elpeleg O. Mitochondrial complex I deficiency caused by a deleterious NDUFA11 mutation. Annals of neurology. 2008;63(3):405-8.

31. Dunning CJ, McKenzie M, Sugiana C, Lazarou M, Silke J, Connelly A, et al. Human CIA30 is involved in the early assembly of mitochondrial complex I and mutations in its gene cause disease. The EMBO journal. 2007;26(13):3227-37.

32. Loeffen J, Elpeleg O, Smeitink J, Smeets R, Stockler-Ipsiroglu S, Mandel H, et al. Mutations in the complex I NDUFS2 gene of patients with cardiomyopathy and encephalomyopathy. Annals of neurology. 2001;49(2):195-201.

33. Budde SM, van den Heuvel LP, Janssen AJ, Smeets RJ, Buskens CA, DeMeirleir L, et al. Combined enzymatic complex I and III deficiency associated with mutations in the nuclear encoded NDUFS4 gene. Biochemical and biophysical research communications. 2000;275(1):63-8.

34. Loeffen J, Smeitink J, Triepels R, Smeets R, Schuelke M, Sengers R, et al. The first nuclear-encoded complex I mutation in a patient with Leigh syndrome. American journal of human genetics. 1998;63(6):1598-608.

35. Lee TM, Addonizio LJ, Barshop BA, Chung WK. Unusual presentation of propionic acidaemia as isolated cardiomyopathy. Journal of inherited metabolic disease. 2009;32 Suppl 1:S97-101.

36. Alston CL, Ceccatelli Berti C, Blakely EL, Olahova M, He L, McMahon CJ, et al. A recessive homozygous p.Asp92Gly SDHD mutation causes prenatal cardiomyopathy and a severe mitochondrial complex II deficiency. Human genetics. 2015;134(8):869-79.

37. Ohkuma A, Noguchi S, Sugie H, Malicdan MC, Fukuda T, Shimazu K, et al. Clinical and genetic analysis of lipid storage myopathies. Muscle & nerve. 2009;39(3):333-42.

38. Iacobazzi V, Invernizzi F, Baratta S, Pons R, Chung W, Garavaglia B, et al. Molecular and functional analysis of SLC25A20 mutations causing carnitine-acylcarnitine translocase deficiency. Human mutation. 2004;24(4):312-20.

39. Korver-Keularts IM, de Visser M, Bakker HD, Wanders RJ, Vansenne F, Scholte HR, et al. Two Novel Mutations in the SLC25A4 Gene in a Patient with Mitochondrial Myopathy. JIMD reports. 2015;22:39-45.

40. Wedatilake Y, Brown RM, McFarland R, Yaplito-Lee J, Morris AA, Champion M, et al. SURF1 deficiency: a multi-centre natural history study. Orphanet journal of rare diseases. 2013;8:96.

41. Powell CA, Kopajtich R, D'Souza AR, Rorbach J, Kremer LS, Husain RA, et al. TRMT5 Mutations Cause a Defect in Post-transcriptional Modification of Mitochondrial tRNA Associated with Multiple Respiratory-Chain Deficiencies. American journal of human genetics. 2015;97(2):319-28.

42. Shahni R, Wedatilake Y, Cleary MA, Lindley KJ, Sibson KR, Rahman S. A distinct mitochondrial myopathy, lactic acidosis and sideroblastic anemia (MLASA) phenotype associates with YARS2 mutations. American journal of medical genetics Part A. 2013;161a(9):2334-8.
